# Associations Between Prenatal Phthalate Exposure and Atopic Symptoms in Childhood: Effect Modification by Child Sex

**DOI:** 10.3390/toxics13090749

**Published:** 2025-09-03

**Authors:** Khushbu Dharmendra Bhatt, Shachi Mistry, Héctor Lamadrid-Figueroa, Marcela Tamayo-Ortiz, Adriana Mercado-Garcia, Jamil M. Lane, Martha M. Téllez-Rojo, Robert O. Wright, Rosalind J. Wright, Guadalupe Estrada-Gutierrez, Kecia N. Carroll, Cecilia S. Alcala, Maria José Rosa

**Affiliations:** 1 Department of Environmental Medicine, Icahn School of Medicine at Mount Sinai, New York, NY 10029, USA; khushbudharmendra.bhatt@icahn.mssm.edu (K.D.B.); shachi.mistry@mssm.edu (S.M.); jamil.lane@mssm.edu (J.M.L.); robert.wright@mssm.edu (R.O.W.); rosalind.wright@mssm.edu (R.J.W.); kecia.carroll@mssm.edu (K.N.C.); maria.rosa@mssm.edu (M.J.R.); 2 Department of Perinatal Health, Center for Population Health Research, National Institute of Public Health (INSP), Cuernavaca 62100, Mexico; hlamadrid@insp.mx; 3 Department of Environmental Health Sciences, Columbia University Mailman School of Public Health, New York, NY 10032, USA; mt3743@cumc.columbia.edu; 4 Center for Nutrition and Health Research, National Institute of Public Health, Cuernavaca 62100, Mexico; adrianam@insp.mx (A.M.-G.); mmtellez@insp.mx (M.M.T.-R.); 5 Department of Pediatrics, Icahn School of Medicine at Mount Sinai, New York, NY 10029, USA; 6 Institute for Exposomic Research, Icahn School of Medicine at Mount Sinai, New York, NY 10029, USA; 7 Department of Immunobiochemistry, National Institute of Perinatology, Mexico City 11000, Mexico; guadalupe.estrada@inper.gob.mx

**Keywords:** atopic diseases, atopic dermatitis, allergic rhinitis, phthalates, environment, allergy

## Abstract

Background: The global rise in atopic diseases, like atopic dermatitis and allergic rhinitis, may be linked to prenatal exposure to endocrine-disrupting chemicals like phthalates, with potential sex-specific effects. Methods: We analyzed 558 mother–child pairs from the PROGRESS birth cohort in Mexico City. Maternal urinary phthalate metabolites were measured during the 2nd and 3rd trimesters. Atopic dermatitis and allergic rhinitis symptoms were assessed at ages 4–6 and 6–8 years using the International Study of Asthma and Allergies in Childhood survey. Weighted Quantile Sum Regression (WQS) was used to assess sex-specific mixture associations. Individual sex-specific phthalate associations were examined using modified Poisson models with inclusion of product terms and stratification. Models were adjusted for maternal age, education, parity, pre-pregnancy body mass index, and prenatal tobacco exposure. Results: We found that child sex modified associations between the 2nd trimester phthalate mixture and current atopic dermatitis symptoms at both 4–6 years (WQS*sex OR: 1.23, 95% CI: 1.00–1.60) and 6–8 years (WQS*sex OR: 1.46, 95% CI: 1.01–2.10). Among males, higher phthalate concentrations were positively associated with symptoms at both ages (OR: 1.10, 95% CI: 0.92, 1.32; OR: 1.16, 95% CI: 0.92, 1.46), while associations were negative in females (OR: 0.87, 95% CI: 0.73, 1.04; OR: 0.79, 95% CI: 0.62, 1.02). No sex-specific associations were found for 3rd trimester exposures. Individual metabolite analyses also showed effect modification by sex for 2nd trimester exposures. Conclusions: Prenatal exposure to phthalates is associated with atopic dermatitis symptoms in childhood in a sex-specific manner.

## 1. Introduction

The prevalence of atopic diseases has risen in both developed and developing countries with approximately 700 million affected individuals worldwide over the past century [1,2,3]. Atopic diseases like atopic dermatitis and allergic rhinitis adversely influence an individual’s quality of life and increase the rate of comorbid conditions [4]. These diseases are also more prevalent in children compared to adults [2,5]. There is evidence that atopic disease development and exacerbation are influenced by environmental exposures beginning in utero [6,7,8,9]. Atopic dermatitis and allergic rhinitis are part of the atopic march, known as the natural progression of atopic diseases starting in infancy [10,11]. Early life diagnoses of atopic dermatitis and allergic rhinitis are risk factors for subsequent development of respiratory diseases like asthma [12]. Therefore, identifying modifiable risk factors in early life can enhance our understanding of the link between environmental exposures and the atopic march while also informing the optimal timing for public health interventions.

Phthalates are commonly used plasticizers, found in numerous industrial products, cosmetic additives, food packaging, toys, containers, shower curtains, and cleaning and building supplies [13,14]. Individuals are exposed to phthalates daily via diet, inhalation, lactation, and skin contact [15]. Additionally, phthalate metabolites can pass through the placenta and have been found in umbilical cord blood, amniotic fluid, placental tissue, and neonatal meconium [15,16]. Because phthalates are commonly used in everyday life, human exposure to them has become a major issue, particularly among vulnerable populations such as pregnant women and babies [13]. Phthalates have been identified as endocrine disrupting chemicals, substances that disrupt normal hormone action [17] and have been linked to numerous health outcomes, including impaired child cognition, increased respiratory health outcomes, and reduced lung function [15,18,19,20,21,22]. Previous studies have reported mixed results in the association between phthalate exposure during pregnancy and atopic diseases in children, showing either an increased risk or no association at all [13,23,24]. The majority of previous epidemiologic research has focused on risk estimates of single phthalate exposure overlooking the effects of phthalate mixtures that occur in real-world contexts [25,26]. Furthermore, susceptibility to both phthalate exposure and atopic disease is sexually dimorphic [27,28] and has not been explored in the context of mixtures exposure. Understanding the relationship between prenatal phthalate exposure and the development of atopic symptoms in childhood is critical due to the widespread use of phthalates, increasing prevalence of allergic diseases, and the vulnerability of the developing immune system during this stage of development.

Therefore, we sought to address this research gap by investigating sex-specific associations between prenatal exposures to multiple phthalates, individually and as a mixture, and atopic dermatitis and allergic rhinitis symptoms in children aged 4–6 to 6–8 years. We hypothesize that higher prenatal phthalate exposure will be associated with a higher risk of atopic dermatitis and allergic rhinitis symptoms in children in a sex-specific manner.

## 2. Materials and Methods

### 2.1. Study Participants

We leveraged a prospective longitudinal birth cohort called PROGRESS (Programming Research in Obesity, Growth, Environment, and Social Stressors) to achieve the aims of this study. Participants were women who were enrolled in health and prenatal care insurance programs run by the Mexican Social Security System (IMSS) between 2007 and 2011 [29]. The study’s inclusion criteria were as follows: 2nd trimester (<20 weeks of gestation), aged 18 years or older, intending to reside in Mexico City for at least 3 years, having access to a phone, no history of heart or renal issues, no daily alcohol use, singleton births, and no previous use of steroid or anti-epilepsy medication. There were 948 live births and 681 mothers who were actively monitored. For these analyses, 558 mother–child pairs had complete data on exposures, outcomes, and covariates (Figure S1). Institutional review boards from the Icahn School of Medicine at Mount Sinai and the Mexican National Institute of Public Health authorized protocols. All participants provided written informed consent.

### 2.2. Prenatal Urinary Phthalate Metabolite Concentrations

Phthalate metabolites were measured from spot urine samples collected during the 2nd and 3rd trimester of pregnancy. Spot urine samples were stored at −80 °C in 2 mL portions in phthalate-free tubes. The samples were analyzed for the following 15 metabolites: mono-n-butyl phthalate (MBP), mono-isobutyl phthalate (MiBP), mono-hydroxy butyl phthalate (MHBP), mono-hydroxyisobutyl phthalate (MHiBP), mono-3-carboxypropyl phthalate (MCPP), monoethyl phthalate (MEP), mono-2-ethyl-5-carboxypentyl phthalate (MECPP), mono-2-ethylhexyl phthalate (MEHP), mono-2-ethyl-5-hydroxyhexyl phthalate (MEHHP), mono-2-ethyl-5-oxohexyl phthalate (MEOHP), monobenzyl phthalate (MBzP), mono (carboxy-isononyl) phthalate (MCNP) mono(carboxy-isooctyl) phthalate (MCOP), monooxononyl phthalate (MONP), mono-2-ethyl-5-carboxypentyl terephthalate (MECPTP) [30]. In order to reduce dimensionality and address the high correlations among phthalate metabolites derived from the same parent compound, we used molar sums of related metabolites as in previous work [22]. These include ∑DEHP = MEHHP + MEHP + MECPP + MEOHP; ∑DiNP = MONP + MCOP; ∑DiBP = MiBP + MHiBP; and ∑DBP = MHBP + MBP. Phthalate metabolites were measured by the Centers for Disease Control and Prevention using isotope dilution high-performance liquid chromatography with tandem mass spectrometry [31]. Biomarker concentrations were adjusted for urinary specific gravity (SG) to correct for urinary dilution. Specific gravity was determined using a digital handheld refractometer. Urine samples had a median specific gravity of 1.02. The limit of detection of the samples ranged between 0.2 and 0.8 ng/mL.

### 2.3. Atopic Symptoms Assessment

Information on atopic disease symptoms in children were collected during the 4–6 and 6–8 years visits. We used the standardized Spanish version of the International Study of Asthma and Allergies in Childhood (ISAAC) survey [32,33,34,35,36,37,38] to assess the caregiver’s report of atopic dermatitis and allergic rhinitis symptoms [32,33,34,35,36,37,38,39]. For atopic dermatitis symptoms we selected the following: ever atopic dermatitis symptoms were defined as “has your child ever had an itchy rash which was coming and going for at least six months?” Current atopic dermatitis symptoms were defined by report of itchy rash in the past 12 months. For allergic rhinitis we examined the following outcomes: ever allergic rhinitis symptoms were defined as the caregiver reporting yes to the question, “Has your child ever had a problem with sneezing, or a runny, or a blocked nose when he/she DID NOT have a cold or the flu?” Current allergic rhinitis symptoms were defined as a caregiver report of these symptoms in the past 12 months. We also included report of these symptoms in the past 12 months with additionally itchy, watery eyes (current allergic rhinitis symptoms + itchy watery eyes).

### 2.4. Covariates

Covariates were selected based on those previously associated with prenatal phthalate exposure and allergy outcomes, excluding those on the causal pathways and validated factors as shown in the Directed Acyclic Graph (Figure S2). Covariates include parity (primiparous/multiparous), mother age at enrollment (measured continuously in years), educational attainment (no more than high school, high school graduate, >high school), maternal pre-pregnancy body mass index (BMI) (kg/m^2^), and prenatal environmental tobacco smoke (ETS) (yes/no). Only 200 participants in our sample reported smoking during pregnancy; therefore, report of a smoker in the home at either the 2nd or the 3rd trimester of pregnancy was included as a covariate instead (ETS). Maternal pre-pregnancy BMI was estimated from a linear mixed-effects model as previously described [40]. Child’s sex (male/female) was recorded at delivery.

### 2.5. Statistical Analysis

Summary statistics were calculated for all atopic outcomes and covariates, and the distribution of continuous covariates and exposures was examined through visual inspection of histograms. Summary statistics were reported as mean and standard deviation (SD) for continuous variables and *n* (%) for categorical variables. Phthalate metabolite concentrations were log2 transformed. To assess the combined impact of the mixture of phthalates on our outcomes and potential effect modification, a sex-stratified weighted quantile sum (WQS) regression model was used. The combined impact of a mixture on the outcome in question is provided by a WQS estimate and a weighted index in which the contribution of each constituent phthalate to the mixture association is shown through weights summing up to 100 for each sex stratum. We determined a priori to test associations in the positive direction (i.e., higher odds) associated with atopic outcomes. Models were run using 100 bootstraps and 110 repeated holdouts with a 40/60 split in order to obtain more reliable results [41]. Models were adjusted for covariates listed above. We report mean estimates, 95% confidence intervals (CIs) and mean chemical weights across all bootstraps; *p*-values are not reported, and associations are assessed via CI inspection [41]. For associations of interest, we used the “Busgang criteria” to identify relevant contributors to the mixture effect [42,43]. A phthalate was considered a “probable” contributor if at least 90% of the holdouts exceeded the 1/c cutoff (where c is the total number of phthalates in the combination). Phthalates were considered “possible contributors if this was true for at least 50% of holdouts [42,43]. In sensitivity analysis, we used modified Poisson regression models to estimate the associations between 2nd and 3rd trimester individual phthalate metabolite concentrations and atopic dermatitis/allergic rhinitis symptoms [44]. Effect modification by sex was examined via inclusion of product terms (sex*phthalate) and stratification. Models were stratified if the product term *p*-value was <0.05. All analysis were performed using R version 3.5.1 (R Foundation for Statistical Computing, Vienna, Austria) and the “gWQS” package version 3.0.5 was used for mixture analysis [23]. The threshold for statistical significance was set at *p* < 0.05.

## 3. Results

### 3.1. Study Population Characteristics

Overall and sex-stratified distributions of outcomes as well as covariates are shown in Table 1, and the characteristics of phthalate metabolites are shown in Table 2. Sex was approximately evenly split. The average enrollment age of the mothers was 28 years old. Most of the participants were primiparous (60.8%) and had less than a high school education (40.3%) at enrollment. In the 4–6 years evaluation, caregivers noted that 11.6% of the kids had ever atopic dermatitis symptoms, 10.4% had current atopic dermatitis symptoms, 41.8% had ever allergic rhinitis symptoms, 38.9% had current allergic rhinitis symptoms, and 11.6% had current allergic rhinitis symptoms with itchy/watery eyes. Among children aged 6–8, the following atopic outcomes were found: (a) 6.1% had ever atopic dermatitis symptoms, (b) 4.7% had current atopic dermatitis symptoms, (c) 31.2% had ever allergic rhinitis symptoms, (d) 28.5% had current allergic rhinitis symptoms and, (e) 12.2% had current allergic rhinitis symptoms with itchy/watery eyes. Correlations between 2nd and 3rd trimester phthalate metabolite concentrations are shown in Supplemental Figure S3. We saw moderate-to-high phthalate correlations in the same trimester and weak correlations across the trimesters (Figure S3).

### 3.2. Sex-Stratified WQS Analysis

**Figure 1:**
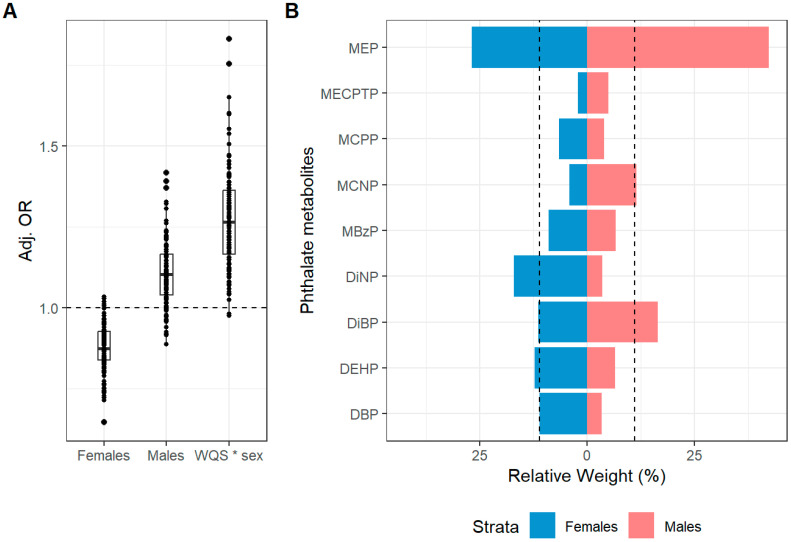
Mean adjusted betas (**A**) and sex-specific relative weights (**B**) from a WQS (positive constraint) linear regression model with 110 repeated holdouts between 2nd trimester phthalates mixture and current atopic dermatitis symptoms at 4–6 years. The model was adjusted for maternal age, BMI, ETS, education and parity. (**A**) Illustrates the distribution of the adjusted betas across the 110 repeated holdouts where each dot represents the estimate from each holdout. (**B**) Illustrates the mean estimated relative weight for each chemical of the phthalate mixtures across the 110 repeated holdouts. The relative weight is the percentage of weight attributable to each chemical in the phthalate mixtures within the total weight of each strata (males and females). The dotted line represents the threshold (11.1%) for chemicals of concern. Chemicals with relative weights above this threshold in at least 50% of the repeated holdouts were considered chemicals of concern. Notes: All chemicals were log2 transformed to reduce skewness in the distribution of the concentrations.

**Figure 2:**
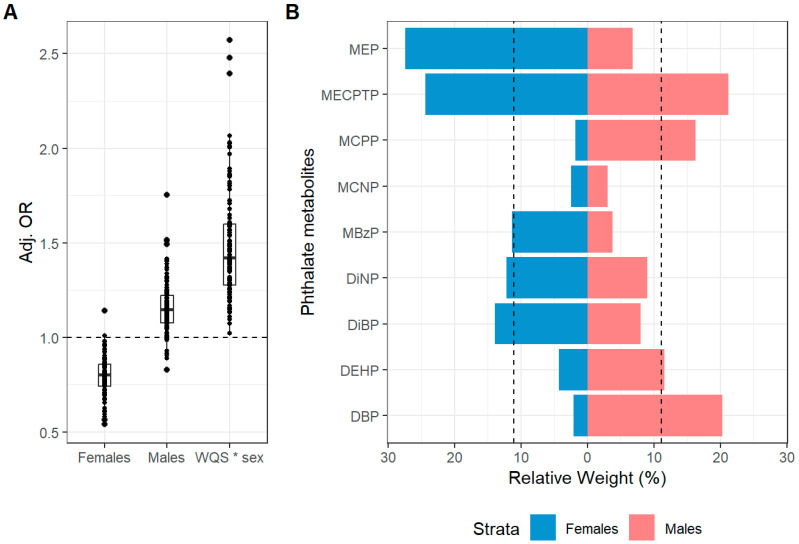
Mean adjusted betas (**A**) and sex-specific relative weights (**B**) from a WQS (positive constraint) linear regression model with 110 repeated holdouts between 2nd trimester phthalates mixture and current atopic dermatitis symptoms at 6–8 years. The model was adjusted for maternal age, BMI, ETS, education and parity. (**A**) Illustrates the distribution of the adjusted betas across the 110 repeated holdouts where each dot represents the estimate from each holdout. (**B**) Illustrates the mean estimated relative weight for each chemical of the phthalate mixtures across the 110 repeated holdouts. The relative weight is the percentage of weight attributable to each chemical in the phthalate mixtures within the total weight of each strata (males and females). The dotted line represents the threshold (11.1%) for chemicals of concern. Chemicals with relative weights above this threshold in at least 50% of the repeated holdouts were considered chemicals of concern. Notes: All chemicals were log2 transformed to reduce skewness in the distribution of the concentrations.

We observed evidence of effect modification by sex on the association between 2nd trimester phthalate metabolites and current atopic dermatitis symptoms at both 4–6 and 6–8 years of age (Figure 1 and Figure 2 and Table 3 and Table 4). The WQS*sex interaction terms (OR: 1.23, 95% CI: 1.00, 1.60 and OR: 1.46, 95% CI: 1.01, 2.10) indicated divergent slopes for males and females. The association between 2nd trimester phthalates mixture and current atopic dermatitis symptoms was in the positive direction for males (OR: 1.10, 95% CI: 0.92, 1.32) with 93/110 holdouts having an OR > 1 while the association was in the negative direction for females (OR: 0.87, 95% CI: 0.73, 1.04) with only 6/110 holdouts having an OR > 1. For males, MEP and ∑DiBP contributed the most to the mixture, while MEP, ∑DiNP and ∑DEHP were the drivers for the female stratum. We saw a similar interaction between 2nd trimester phthalates mixture and sex on current atopic dermatitis symptoms at 6–8 years. This association was in the positive direction in males (OR: 1.16, 95% CI: 0.92, 1.46) with 99/110 holdouts having an OR > 1 while in females the association was in the negative direction (OR: 0.79, 95% CI: 0.62, 1.02) with only 2/110 holdouts having an OR > 1. For males, MECPTP, ∑DBP, MCPP and ∑DEHP contributed the most to the mixture, whereas MEP, MECPTP, ∑DiBP, ∑DiNP and MBzP were the drivers in females. We did not find evidence of sex-specific associations between 2nd trimester phthalate mixture and ever atopic dermatitis or any allergic rhinitis symptoms (Supplemental Figures S4–S11, Supplemental Tables S1–S8). We also did not find any evidence of sex-specific mixture associations between 3rd trimester phthalate concentrations and any atopic disease outcome (Supplemental Figures S12–S21, Supplemental Tables S9–S18). However, in general, we saw similar patterns to those observed with 2nd trimester concentrations with positive (i.e., higher odds) of dermatitis symptoms in males and negative associations in females.

### 3.3. Sensitivity Analyses

In individual models for the overall sample, higher 2nd trimester concentrations of MECPTP were associated with lower risk of ever (RR: 0.84, 95% CI: 0.72–0.98) and current atopic dermatitis symptoms (RR: 0.84, 95% CI: 0.71–0.98) at 4–6 years (Supplemental Table S19). Second trimester levels of ∑DEHP (RR: 0.91, 95% CI: 0.84–0.99) and MCPP (RR: 0.90, 95% CI: 0.82–0.99) were associated with lower risk of ever allergic rhinitis symptoms at 6–8 years of age (Supplemental Table S20). Higher 3rd trimester levels of MECPTP were associated with lower risk of ever (RR: 0.84, 95% CI: 0.72–0.97) and current atopic dermatitis symptoms (RR: 0.84, 95% CI: 0.72–0.98) at 4–6 years of age (Supplemental Table S21). Higher 3rd trimester levels of MCPP were associated with lower risk of ever (RR: 0.91, 95%, CI: 0.83–0.97) and current allergic rhinitis symptoms (RR: 0.90, 95% CI: 0.82–0.91) at 6–8 years of age (Supplemental Table S22).

Evidence of effect modification by sex was only seen for 2nd trimester phthalate metabolite concentrations and allergic outcomes (Table S23). We identified effect modification by sex on the association between 2nd trimester levels of MCNP and ever atopic dermatitis symptoms at 4–6 years (*p*-interaction = 0.01). MCNP levels measured during the 2nd trimester were associated with higher risk of ever atopic dermatitis symptoms at 4–6 years in males (RR: 1.16, 95% CI: 0.88, 1.54) and with lower risk in females (RR: 0.67, 95% CI: 0.48, 0.92). Additionally, we observed effect modification by sex on the association between 2nd trimester levels of MCNP and current atopic dermatitis symptoms at 4–6 years (*p*-interaction = 0.02). MCNP levels measured during the 2nd trimester were associated with higher risk of current atopic dermatitis symptoms at 4–6 years in males (RR: 1.21, 95% CI: 0.87, 1.66) and with lower risk in females (RR: 0.70, 95% CI: 0.50, 0.98).

Effect modification by sex was seen on the association between 2nd trimester levels of ∑DBP and ever allergic rhinitis symptoms at 4–6 years (*p*-interaction = 0.05). Levels of ∑DBP measured during the 2nd trimester were associated with reduced risk of ever allergic rhinitis symptoms at 4–6 years in males (RR: 0.98, 95% CI: 0.91, 1.06) and with higher risk in females (RR: 1.11, 95% CI: 1.01, 1.23). Effect modification by sex was seen on the association between 2nd trimester levels of ∑DBP and current allergic rhinitis symptoms at 4–6 years (*p*-interaction = 0.03). ∑DBP levels measured during the 2nd trimester were associated with lower risk of ever allergic rhinitis symptoms at 4–6 years in males (RR: 0.97, 95% CI: 0.89, 1.05) and with higher risk in females (RR: 1.11, 95% CI: 1.00, 1.23). The effect modification by sex was seen on the association between 2nd trimester levels of MCPP and current atopic dermatitis symptoms at 6–8 years (*p*-interaction = 0.03). MCPP levels during the 2nd trimester were associated with higher risk of current atopic dermatitis symptoms at 6–8 years in males (RR: 1.34, 95% CI: 0.86, 2.08) and with lower risk in females (RR: 0.69, 95% CI: 0.47, 1.02).

## 4. Discussion

In this prospective study, we found evidence of sex-specific associations between a 2nd trimester phthalate mixture and risk of current atopic dermatitis symptoms at ages 4–6 and 6–8 years, with patterns of higher risk in males and lower risk in females. Among males, MEP and ∑DiBP were the primary contributors to the mixture association with allergic outcomes at 4–6 years of age, while MECPTP, ∑DBP, MCPP and ∑DEHP were the key contributors at 6–8 years of age. Among females, MEP, ∑DiNP, ∑DEHP were the primary contributors to the mixture association with allergic outcomes at 4–6 years of age, while MEP, MECPTP, ∑DiBP, ∑DiNP and MBzP were the top contributors at 6–8 years of age. We did not find any evidence of sex-specific associations between 2nd trimester phthalate concentrations and allergic rhinitis symptoms or between 3rd trimester phthalate concentrations and any outcome.

This study adds to the growing research on prenatal phthalate exposure and its association with allergic outcomes in children. Casas et al. conducted a review of 19 prospective studies published between 2011 and 2020 focused on the impact of prenatal exposure to non-persistent EDCs on asthma and allergic diseases [45]. The review concluded that there was insufficient evidence for the association of prenatal exposure to phthalates and allergic diseases; however, it did not include any studies from Latin America [45]. In a study of children living in Salinas, California, researchers assessed individual associations between three low molecular weight phthalate metabolites, parabens and phenols measured in pregnancy and multiple allergic and respiratory outcomes in children at age 7 with adjustment for other exposures [24]. The majority of the associations were null; phthalate concentrations were associated with higher levels of Th2 cytokines and lower lung function but were not associated with any atopic disease outcome [24]. In a separate analysis by the same group, they examined high molecular weight phthalates and bisphenol A in pregnancy and the same outcomes at age 7. MCPP concentrations were associated with increased odds for aeroallergies in demographically adjusted models, but the associations were attenuated with adjustment for other chemical concentrations [46]. The Odense child cohort in Denmark reported no consistent associations between prenatal phthalate exposure, individually or as a mixture, and asthma and allergic dermatitis in children at 5 years of age [23]. Even fewer studies have examined whether these associations varied by sex. The EDEN (Etude des Déterminants pré et post natals du dévelopment de la santé de l’Enfant) birth cohort based in France reported positive associations between prenatal exposure to metabolites of ∑DiNP and ∑DiBP and early and late onset eczema in the first 5 years of life in males but not in females [47]. In an analysis using data from the ECHO-PATHWAYS consortium, researchers found an association between 3rd trimester phthalate mixtures and childhood asthma and wheeze at the age of 4–6 years, which was more pronounced in males [48]. Buckley et al. examined the association between prenatal environmental phenol and phthalates during the 3rd trimester and respiratory and allergic diseases in children at age 6–8 years participating in the Mount Sinai Children’s Environmental Health Study [49]. This study found that biomarkers of low molecular weight phthalates, MEP, MnBP and MiBP, were associated with reduced odds of wheeze symptoms, particularly among girls [49]. In this study, MEP was one of the primary contributors to phthalate mixture associations with allergic outcomes at 4–6 years of age among males and females and remained one of the top contributors among females at 6–8 years of age. Our group reported associations between prenatal phthalate metabolite concentrations and wheeze and asthma in PROGRESS children at 4–6 years of age in which mixture associations between 2nd trimester phthalate metabolite concentrations and asthma at 4–6 years of age were stronger in males compared to females [22]. Our results align with previous work suggesting males may be more susceptible to these in utero exposures.

Variability in urinary metabolite concentrations across studies may reflect differences in exposure sources and population characteristics [19]. Our study population had notably higher levels of DEHP, DBP, and MEP compared with cohorts in the US [50], China [51], Canada [52], France [53], and Puerto Rico [54]. Relative to the ELEMENT cohort of pregnant women in Mexico (1997–2005), our participants are also exhibited elevated concentrations of several phthalate metabolites, including MBzP and those derived from DBP, DiBP and DEHP [55]. These elevated exposure levels highlight the importance of considering geographic and sociodemographic context when interpretating phthalate related health effects. The findings of this study contribute to the growing body of literature by demonstrating sex-specific associations in a population with comparatively high phthalate exposures, which offers insight into potential critical windows of susceptibility and broader generalizability of results across diverse settings.

Variability in phthalate concentrations between the 2nd and 3rd trimesters reflects their short biological half-lives as well as changes in maternal behaviors and product use across pregnancy. We observed associations between MEP and the sum of DiBP with allergic outcomes at ages 4–6 years. Notably, MEP remained a leading contributor to these associations at ages 6–8. The presence of associations at 4–6 years, but not 6–8 years, may point to critical windows of susceptibility and potential age-related differences in symptom presentation. Additionally, we observed associations between prenatal exposure to phthalates during the 2nd trimester and increased risk of atopic disease symptoms, whereas no such associations were found for the 3rd trimester exposures. This may reflect critical windows of susceptibility, as the 2nd trimester is a sensitive period for the development of the fetal lungs and immune system. At this time, the fetus is more vulnerable to the disruptive effects of phthalates [21,22]. Particularly, the lungs transition from solid glandular organs to a hollow ones capable of supporting extra-uterine gas exchange [22,56]. This stage of lung development is vulnerable to disruptions in fetal breathing movements, space for lung expansion and lung liquid retention [21,22]. Furthermore, the fetal thyroid gland reaches functional maturity during this time, which may further contribute to susceptibility given the known endocrine disrupting properties of phthalates [57,58].

Prenatal phthalate exposure may influence allergy development through multiple mechanisms, including but not limited to affecting immune function, promoting oxidative stress, and inflammation. Phthalates may alter the endocrine system and disrupt the action of some hormones such as glucocorticoids, which are crucial for the fetal immune system [59,60,61]. For example, glucocorticoid receptor antagonism will affect the generation of dendritic and regulatory T cells that are essential for immune tolerance and tumor control [62]. Phthalates promote oxidative stress by producing reactive oxygen species (ROS) and this creates an imbalance between ROS production and ROS neutralization by the body through anti-oxidative processes [63,64]. Exposure to oxidative stress during the fetal period may affect immune system development, airway formation, and barrier integrity [65]. Impaired epithelial barrier activity in the skin and respiratory system, coupled with inflammation, can increase vulnerability to environmental allergens, potentially leading to allergic reactions.

Sex-specific differences in fetal development may lead to sexually dimorphic effects of phthalate exposure. Phthalates are known to interfere with the synthesis, metabolism, and signaling of estrogen and androgen [21,66]. Androgens could potentially play a role in the occurrence of atopic disease symptoms, as androgens suppress Th1/Th2/Th17 and induce Th2/regulatory T cells [67]. For instance, pregnant women carrying female fetuses exhibit better-regulated immune responses, whereas those with male fetuses show heightened inflammation [68]. Male fetuses also display cytokine profiles with more inflammatory and proangiogenic cytokines, while female fetuses have increased regulatory cytokines [68]. Additionally, global gene expression analysis of placentas by sex reveals greater gene expression in cytotrophoblast cells of male fetuses, highlighting distinct immune development pathways between sexes [68]. Females may be less susceptible to phthalate exposure in the context of allergic disease due to estrogen’s antioxidant properties, which could mitigate oxidative stress [69].

This study has several strengths, including longitudinal follow-up, assessment of phthalate metabolites at two different time points and the use of advanced statistical methods. We were able to obtain sex-specific mixture associations and weights using stratified WQS models. In addition, most biomonitoring studies have primarily focused on populations in the US, Canada, Europe and Asia [14,70], and evidence from Latin America is limited. The identified metabolites of interest are widely prevalent, either present in personal care products or used as plasticizers in commercial items, which are not currently regulated in Mexico. We also acknowledge some limitations. Misclassification bias could potentially be a problem due to the parents and guardians inaccurately reporting their child’s atopic disease status, which would be non-differential, potentially underestimating the effects. Future studies could minimize this by validating outcomes through medical records or physician diagnoses. In addition, we assessed phthalate exposure using spot urine samples collected during the 2nd and 3rd trimesters, which primarily reflect recent exposure. A more comprehensive assessment could be achieved by collecting multiple spot samples at different time points or 24-h urine samples, although this was not feasible within our study design and resources.

## 5. Conclusions

This study suggests that phthalate exposure during pregnancy may be associated with increased atopic outcomes in children, particularly atopic dermatitis. These findings suggest that pregnancy exposure to phthalates has a lasting effect on a child’s immune system and its development after birth and requires follow-up at later ages of the child. Future work will examine if these associations persist into later childhood/adolescence and if they potentially mediate asthma development.

## Figures and Tables

**Table 1:** Characteristics of mother-child dyads in the PROGRESS cohort, *n* = 558.

Characteristic	Total Sample (N = 558)	Females (N = 282)	Males (N = 276)
Maternal age at enrollment (years), Mean (SD)	27.5 (5.54)	27.3 (5.67)	27.7 (5.42)
Maternal education at enrollment, *n* (%)			
<High school	225 (40.3%)	104 (36.9%)	121 (43.8%)
High school	206 (36.9%)	113 (40.1%)	93 (33.7%)
>High school	127 (22.8%)	65 (23.0%)	62 (22.5%)
Parity, *n* (%)			
Primiparous	339 (60.8%)	165 (58.5%)	174 (63.0%)
Multiparous	219 (39.2%)	117 (41.5%)	102 (37.0%)
Child’s age, Mean (SD)			
4–6 years	4.43 (0.567)	4.44 (0.577)	4.42 (0.556)
6–8 years	6.26 (0.556)	6.26 (0.585)	6.25 (0.527)
Report of a smoker in the home during pregnancy			
No	358 (64.2%)	179 (63.5%)	179 (64.9%)
Yes	200 (35.8%)	103 (36.5%)	97 (35.1%)
Maternal pre-pregnancy BMI (kg/m^2^, Mean (SD)	26.4 (4.16)	26.1 (4.22)	26.7 (4.08)
Atopic Outcomes (4–6 years), *n* (%)			
Ever atopic dermatitis symptoms, yes	65 (11.6%)	34 (12.1%)	31 (11.2%)
Current atopic dermatitis symptoms, yes	58 (10.4%)	31 (11.0%)	27 (9.8%)
Ever allergic rhinitis symptoms, yes	233 (41.8%)	100 (35.5%)	133 (48.2%)
Current allergic rhinitis symptoms, yes	217 (38.9%)	95 (33.7%)	122 (44.2%)
Current allergic rhinitis symptoms+ itchy/watery eyes, yes	65 (11.6%)	31 (11.0%)	34 (12.3%)
Atopic Outcomes (6–8 years), *n* (%)			
Ever atopic dermatitis symptoms, yes	34 (6.1%)	16 (5.7%)	18 (6.5%)
Current atopic dermatitis symptoms, yes	26 (4.7%)	13 (4.6%)	13 (4.7%)
Ever allergic rhinitis symptoms, yes	174 (31.2%)	71 (25.2%)	103 (37.3%)
Current allergic rhinitis symptoms, yes	159 (28.5%)	63 (22.3%)	96 (34.8%)
Current allergic rhinitis symptoms + itchy/watery eyes, yes	68 (12.2%)	27 (9.6%)	41 (14.9%)

Abbreviations: BMI (body mass index). ETS (environmental tobacco smoke), smoker in the home is defined as someone in the home who smoked more than 5 cigarettes per day and resided in the home at least 4 days per week [41].

**Table 2:** Characteristics of phthalate metabolites of mother-child dyads in the PROGRESS cohort, *n* = 558.

Characteristic	Total Sample (N = 558)	Females (N = 282)	Males (N = 276)
Phthalate Metabolites during the 2nd Trimester, Mean (SD) (log transformed)			
∑DEHP	6.49 (1.38)	6.51 (1.35)	6.47 (1.41)
MECPTP	0.982 (1.52)	1.00 (1.50)	0.960 (1.55)
∑DiNP	2.62 (1.44)	2.63 (1.48)	2.60 (1.40)
MCNP	−0.0299 (1.20)	−0.0197 (1.23)	−0.0404 (1.17)
MCPP	0.540 (1.28)	0.604 (1.32)	0.475 (1.24)
MBzP	2.47 (1.73)	2.49 (1.66)	2.45 (1.80)
Low molecular weight			
∑DiBP	3.64 (1.34)	3.66 (1.30)	3.61 (1.39)
∑DBP	6.46 (1.49)	6.50 (1.46)	6.42 (1.52)
MEP	7.16 (1.95)	7.12 (1.97)	7.19 (1.94)
Phthalate Metabolites during the 3rd Trimester, Mean (SD) (log transformed)			
High molecular weight			
∑DEHP	6.65 (1.39)	6.62 (1.28)	6.68 (1.50)
Missing	65 (11.6%)	28 (9.9%)	37 (13.4%)
MECPTP	1.26 (1.61)	1.37 (1.66)	1.14 (1.56)
Missing	69 (12.4%)	29 (10.3%)	40 (14.5%)
∑DiNP	2.63 (1.32)	2.59 (1.29)	2.66 (1.35)
Missing	65 (11.6%)	28 (9.9%)	37 (13.4%)
MCNP	−0.00508 (1.14)	−0.0333 (1.16)	0.0249 (1.12)
Missing	65 (11.6%)	28 (9.9%)	37 (13.4%)
MCPP	0.495 (1.31)	0.512 (1.30)	0.477 (1.31)
Missing	67 (12.0%)	28 (9.9%)	39 (14.1%)
MBzP	2.39 (1.67)	2.40 (1.68)	2.37 (1.67)
Missing	69 (12.4%)	31 (11.0%)	38 (13.8%)
Low Molecular weight			
∑DiBP	3.73 (1.41)	3.76 (1.34)	3.71 (1.49)
Missing	65 (11.6%)	28 (9.9%)	37 (13.4%)
∑DBP	6.47 (1.48)	6.45 (1.45)	6.48 (1.52)
Missing	65 (11.6%)	28 (9.9%)	37 (13.4%)
MEP	7.19 (2.03)	7.21 (2.09)	7.17 (1.97)
Missing	65 (11.6%)	28 (9.9%)	37 (13.4%)

Abbreviations: Di-2-ethylhexyl phthalate (DEHP), Diisononyl phthalate (DiNP), Diisobutyl phthalate (DiBP), Dibutyl phthalate (DBP), mono-2-ethyl-5-carboxypentyl terephthalate (MECPTP), mono (carboxy-isononyl) phthalate (MCNP), mono-3-carboxypropyl phthalate (MCPP), monobenzyl phthalate (MBzP), monoethyl phthalate (MEP).

**Table 3:** Mean adjusted association from WQS (positive constraint) binomial model with 110 repeated holdouts between 2nd trimester phthalates mixture and current atopic dermatitis symptoms at 4–6 years of age (N = 558).

Current Atopic Dermatitis Symptoms ^a^	Mean OR and 95% CI ^b^	OR > 1 ^c^
WQS (b_1_)	0.87 (0.73, 1.04)	6/110
WQS*sex (b_12_)	1.23 (1.00, 1.60)	106/110
Betas for males and females		
Females (b_1_) ^d^	0.87 (0.73, 1.04)	6/110
Males (b_2_) ^d^	1.10 (0.92, 1.32)	93/110

Abbreviations: WQS (weighted quantile sum), OR (Odds Ratio), CI (confidence interval). All compounds underwent log2 transformation to decrease skewness in the concentration distribution. (^a^) The association is based on a stratified Weighted Quantile Sum (WQS) linear regression model that incorporates sex-specific weights and features an interaction term WQS*sex. The model was executed using 110 repeated holdouts, utilizing 40% of the data for training and 60% for validation. The models were adjusted for maternal age, pre-pregnancy BMI, exposure to environmental tobacco smoke during pregnancy, educational attainment at enrollment, and parity. (^b^) The average beta throughout the 110 repeated holdouts is displayed along with its 95% confidence interval. (^c^) The quantity of corrected betas from the 110 repeated holdouts that were positive. (^d^) The adjusted beta for females (b_1_) represents the beta for the WQS index within the reference group (females = 0), while the adjusted beta for males (b_2_), the comparison group (males = 1), is determined by the sum of the beta for the WQS*sex interaction term and the beta for the WQS index for the reference group (b_2_ = b_1_ + b_12_).

**Table 4:** Mean adjusted association from WQS (positive constraint) binomial model with 110 repeated holdouts between 2nd trimester phthalates mixture and current atopic dermatitis symptoms at 6–8 years of age (N = 558).

Current Atopic Dermatitis Symptoms ^a^	Mean OR and 95% CI ^b^	OR > 1 ^c^
WQS (b_1_)	0.79 (0.62, 1.02)	2/110
WQS*sex (b_12_)	1.46 (1.01, 2.1)	109/110
Betas for males and females		
Females (b_1_) ^d^	0.79 (0.62, 1.02)	2/110
Males (b_2_) ^d^	1.16 (0.92, 1.46)	99/110

Abbreviations: WQS (weighted quantile sum), OR (Odds Ratio), CI (confidence interval). All compounds underwent log2 transformation to decrease skewness in the concentration distribution. (^a^) The association is based on a stratified Weighted Quantile Sum (WQS) linear regression model that incorporates sex-specific weights and features an interaction term WQS*sex. The model was executed using 110 repeated holdouts, utilizing 40% of the data for training and 60% for validation. The models were modified for mother age, pre-pregnancy BMI, exposure to environmental tobacco smoke during pregnancy, educational attainment at enrollment, and parity. (^b^) The average beta throughout the 110 repeated holdouts is displayed along with its 95% confidence interval. (^c^) The quantity of corrected betas from the 110 repeated holdouts that were positive. (^d^) The adjusted beta for females (b_1_) represents the beta for the WQS index within the reference group (females = 0), while the adjusted beta for males (b_2_), the comparison group (males = 1), is determined by the sum of the beta for the WQS*sex interaction term and the beta for the WQS index for the reference group (b_2_ = b_1_ + b_12_).

## Data Availability

The data presented in this study are available on request from the corresponding author due to ethical reasons.

## Supplementary Materials

The following supporting information can be downloaded at: https://www.mdpi.com/article/10.3390/toxics13090749/s1.
