# Supplementary material for: Associations Between Prenatal Phthalate Exposure and Atopic Symptoms in Childhood: Effect Modification by Child Sex
Source: Toxics. 2025 Sep 3;13(9):749. doi: 10.3390/toxics13090749 (PMC12473533; doi:10.3390/toxics13090749)
Supplement: Supplementary file 1 [file toxics-13-00749-s001.zip › toxics-3797991-supplementary.pdf]

Supplementary Materials

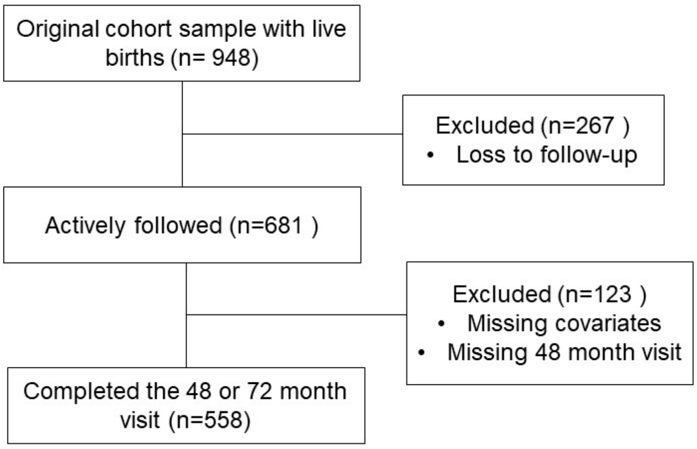

Figure S1. Flow diagram of participants included in analysis.

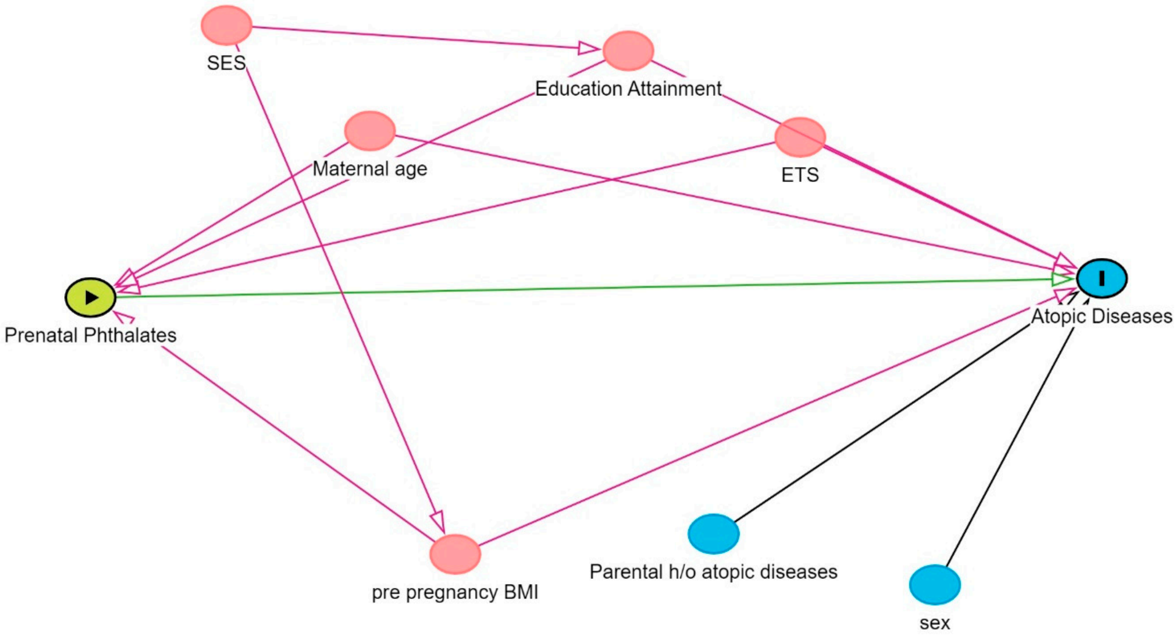

Figure S2. Directed Acyclic Graph (DAG) of assumed dependencies between prenatal exposure to phthalates and childhood atopic symptoms, with other health-related factors.

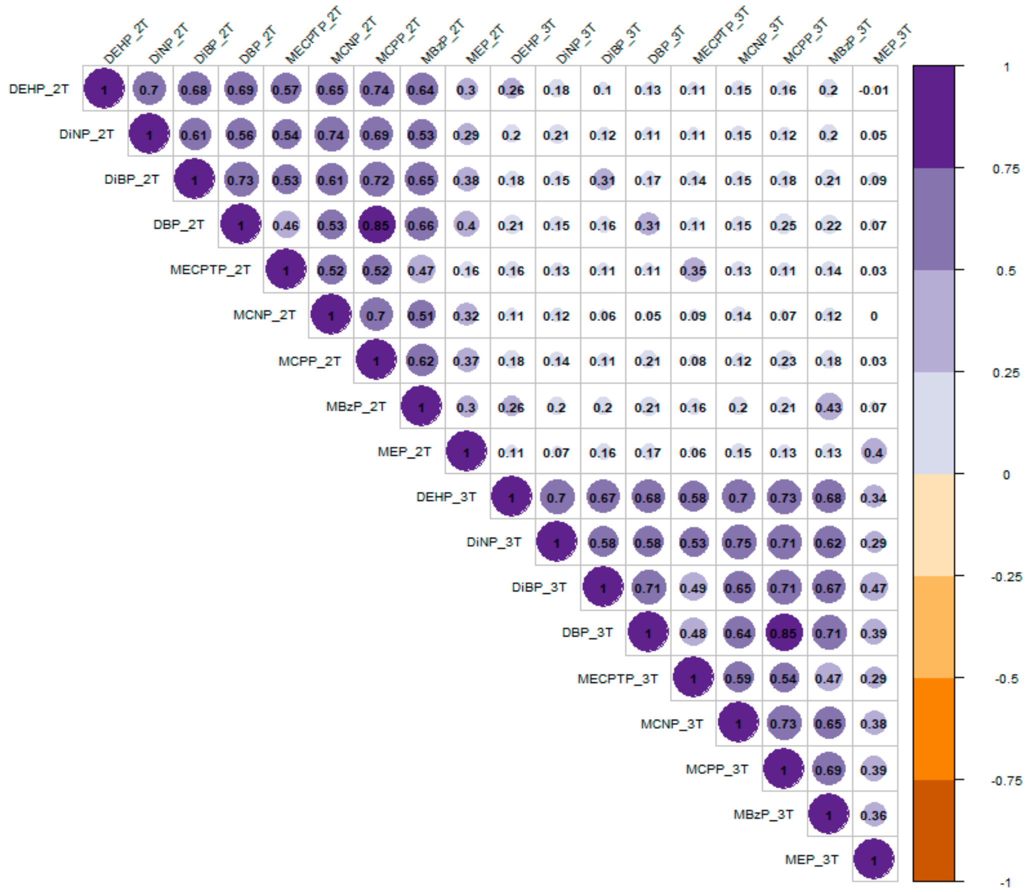

Figure S3. Pearson correlations between log2 transformed 2nd trimester and 3rd trimester phthalate metabolite concentrations

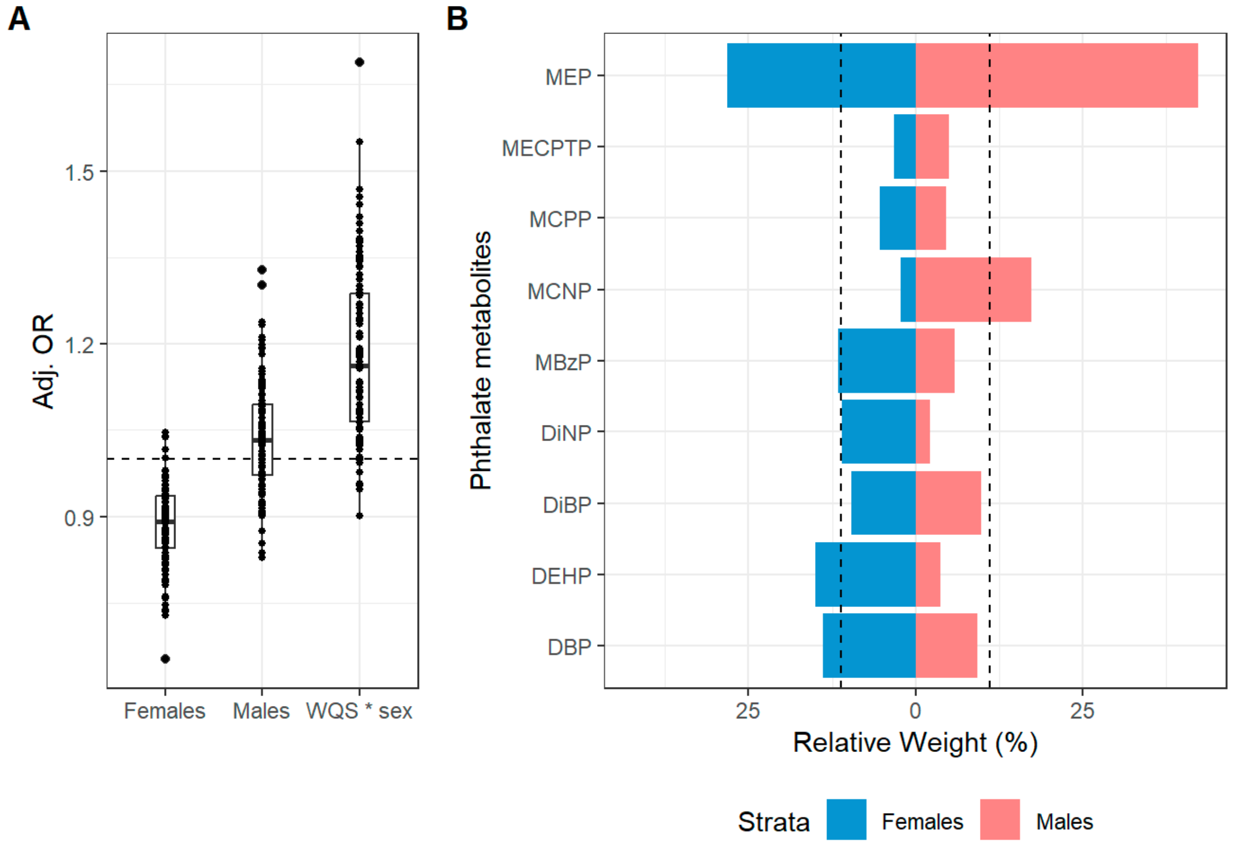

Figure S4. Mean adjusted betas (A) and sex-specific relative weights (B) from a WQS (positive constraint) linear regression model with 110 repeated holdouts between 2nd trimester phthalates mixture and ever atopic dermatitis symptoms at 4-6 years. The model was adjusted for maternal age, BMI, ETS, education, parity. (A) Illustrates the distribution of the adjusted betas across the 110 repeated holdouts where each dot represents the estimate from each holdout. (B) Illustrates the mean estimated relative weight for each chemical of the phthalate mixtures across the 110 repeated holdouts. The relative weight is the percentage of weight attributable to each chemical in the phthalate mixtures within the total weight of each strata (males and females). The dotted line represents the threshold (11.1%) for chemicals of concern. Chemicals with relative weights above this threshold in at least 50% of the repeated holdouts were considered chemicals of concern. Abbreviations: BMI = body mass index; ETS = environmental tobacco smoke; Notes: All chemicals were log_2 transformed to reduce skewness in the distribution of the concentrations.

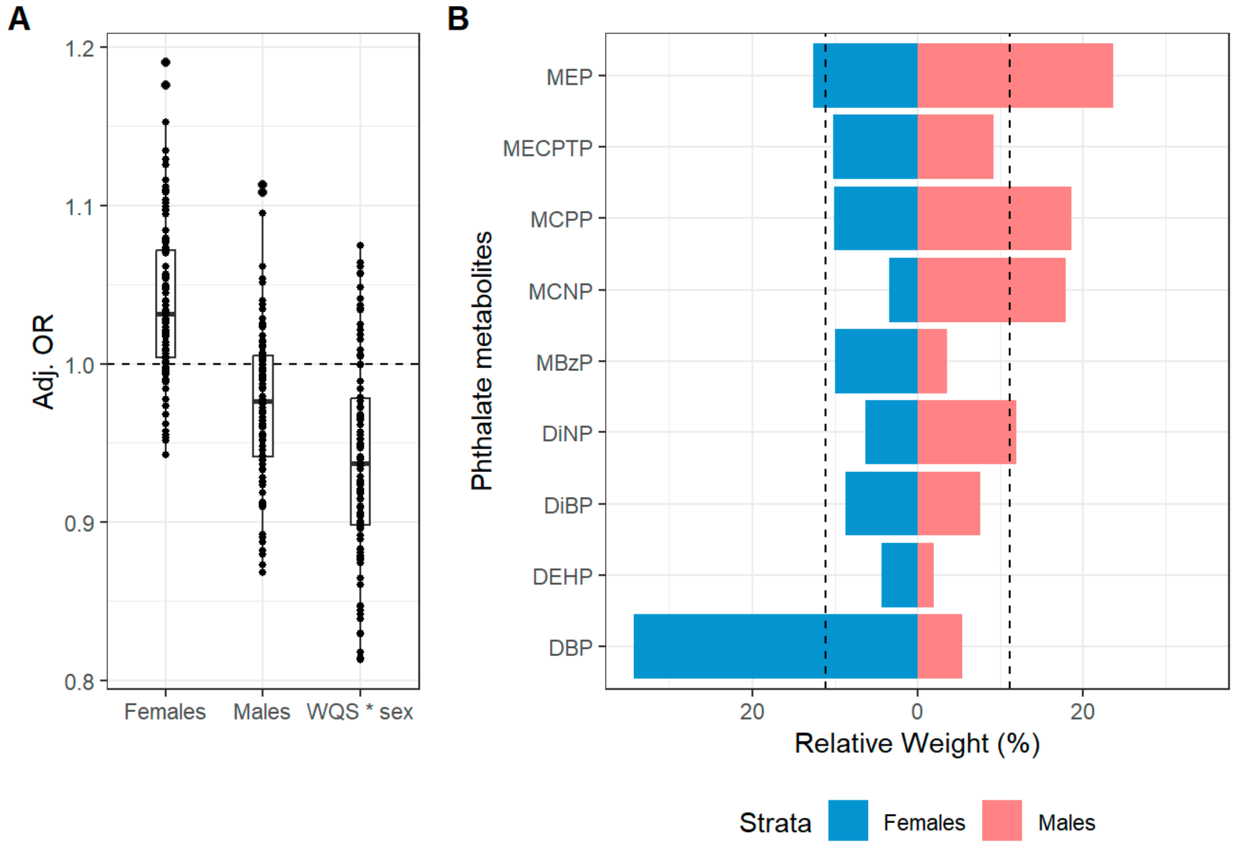

Figure S5. Mean adjusted betas (A) and sex-specific relative weights (B) from a WQS (positive constraint) linear regression model with 110 repeated holdouts between 2nd trimester phthalates mixture and ever allergic rhinitis symptoms at 4-6 years. The model was adjusted for maternal age, BMI, ETS, education, parity. (A) Illustrates the distribution of the adjusted betas across the 110 repeated holdouts where each dot represents the estimate from each holdout. (B) Illustrates the mean estimated relative weight for each chemical of the phthalate mixtures across the 110 repeated holdouts. The relative weight is the percentage of weight attributable to each chemical in the phthalate mixtures within the total weight of each strata (males and females). The dotted line represents the threshold (11.1%) for chemicals of concern. Chemicals with relative weights above this threshold in at least 50% of the repeated holdouts were considered chemicals of concern. Abbreviations: BMI = body mass index; ETS = environmental tobacco smoke; Notes: All chemicals were $\log_2$ transformed to reduce skewness in the distribution of the concentrations.

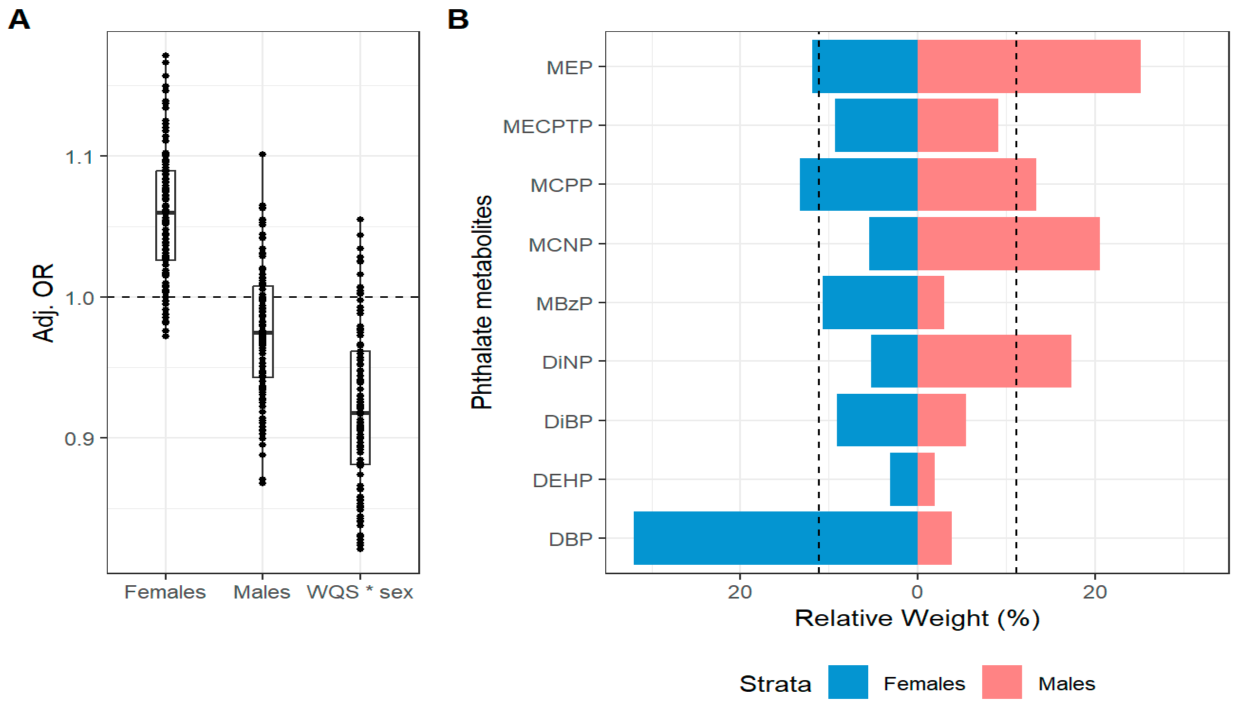

Figure S6. Mean adjusted betas (A) and sex-specific relative weights (B) from a WQS (positive constraint) linear regression model with 110 repeated holdouts between 2nd trimester phthalates mixture and current allergic rhinitis symptoms at 4-6 years. The model was adjusted for maternal age, BMI, ETS, education, parity. (A) Illustrates the distribution of the adjusted betas across the 110 repeated holdouts where each dot represents the estimate from each holdout. (B) Illustrates the mean estimated relative weight for each chemical of the phthalate mixtures across the 110 repeated holdouts. The relative weight is the percentage of weight attributable to each chemical in the phthalate mixtures within the total weight of each strata (males and females). The dotted line represents the threshold (11.1%) for chemicals of concern. Chemicals with relative weights above this threshold in at least 50% of the repeated holdouts were considered chemicals of
concern. Abbreviations: BMI = body mass index; ETS = environmental tobacco smoke; Notes: All chemicals were $\log_2$ transformed to reduce skewness in the distribution of the concentrations.

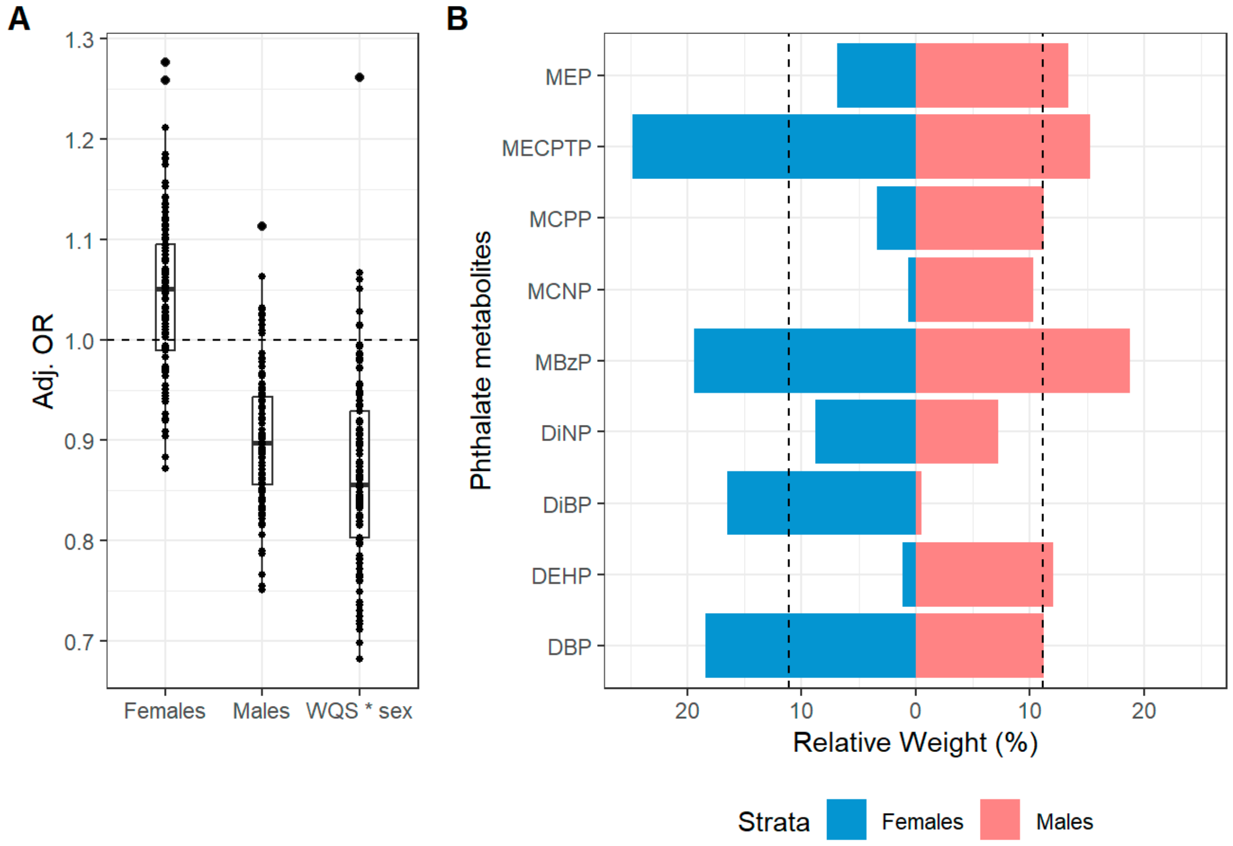

Figure S7. Mean adjusted betas (A) and sex-specific relative weights (B) from a WQS (positive constraint) linear regression model with 110 repeated holdouts between 2nd trimester phthalates mixture and current allergic rhinitis symptoms + itchy watery eyes at 4-6 years. The model was adjusted for maternal age, BMI, ETS, education, parity. (A) Illustrates the distribution of the adjusted betas across the 110 repeated holdouts where each dot represents the estimate from each holdout. (B) Illustrates the mean estimated relative weight for each chemical of the phthalate mixtures across the 110 repeated holdouts. The relative weight is the percentage of weight attributable to each chemical in the phthalate mixtures within the total weight of each strata (males and females). The dotted line represents the threshold (11.1%) for chemicals of concern. Chemicals with relative weights above this threshold in at least 50% of the repeated holdouts were considered chemicals of concern. Abbreviations: BMI = body mass index; ETS = environmental tobacco smoke; Notes: All chemicals were $\log_2$ transformed to reduce skewness in the distribution of the concentrations.

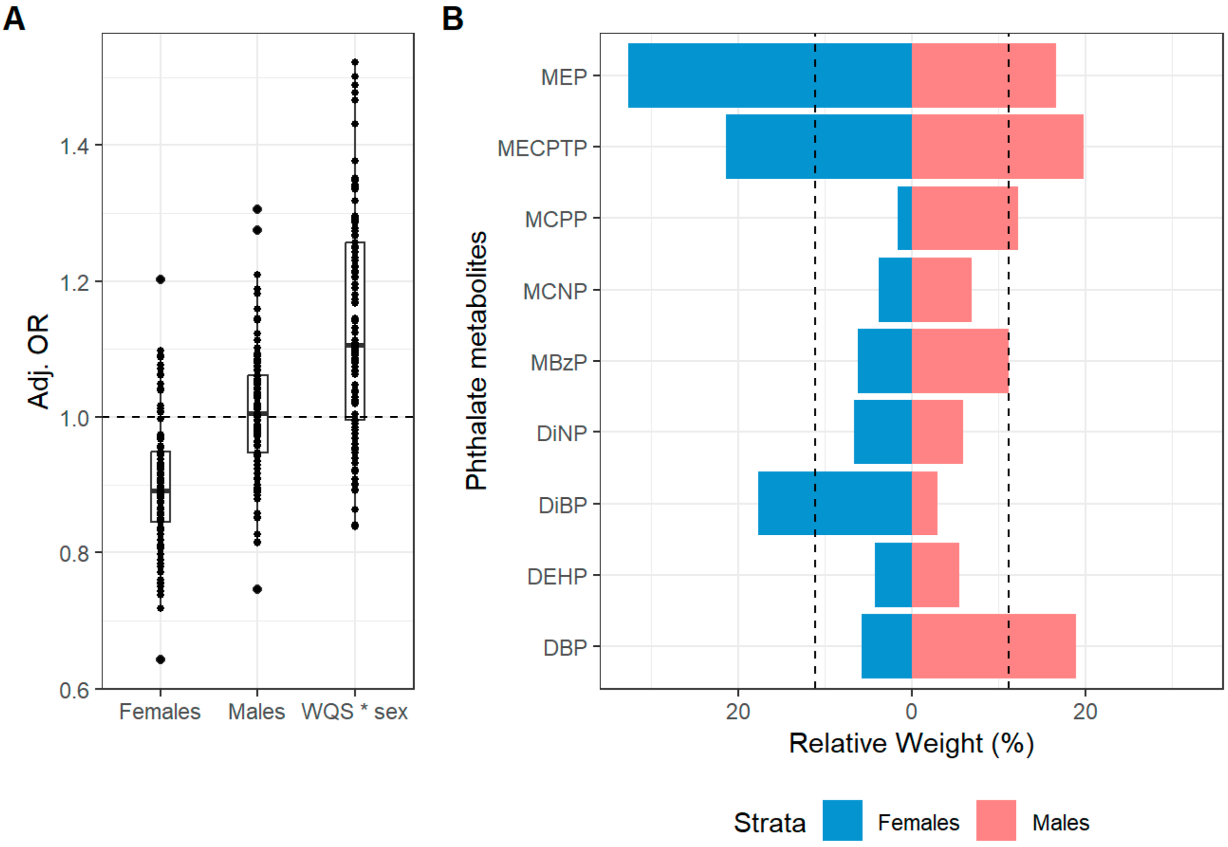

Figure S8. Mean adjusted betas (A) and sex-specific relative weights (B) from a WQS (positive constraint) linear regression model with 110 repeated holdouts between 2nd trimester phthalates mixture and ever atopic dermatitis symptoms at 6-8 years. The model was adjusted for maternal age, BMI, ETS, education, parity. (A) Illustrates the distribution of the adjusted betas across the 110 repeated holdouts where each dot represents the estimate from each holdout. (B) Illustrates the mean estimated relative weight for each chemical of the phthalate mixtures across the 110 repeated holdouts. The relative weight is the percentage of weight attributable to each chemical in the phthalate mixtures within the total weight of each strata (males and females). The dotted line represents the threshold (11.1%) for chemicals of concern. Chemicals with relative weights above this threshold in at least 50% of the repeated holdouts were considered chemicals of concern. Abbreviations: BMI = body mass index; ETS = environmental tobacco smoke; Notes: All chemicals were $\log_2$ transformed to reduce skewness in the distribution of the concentrations.

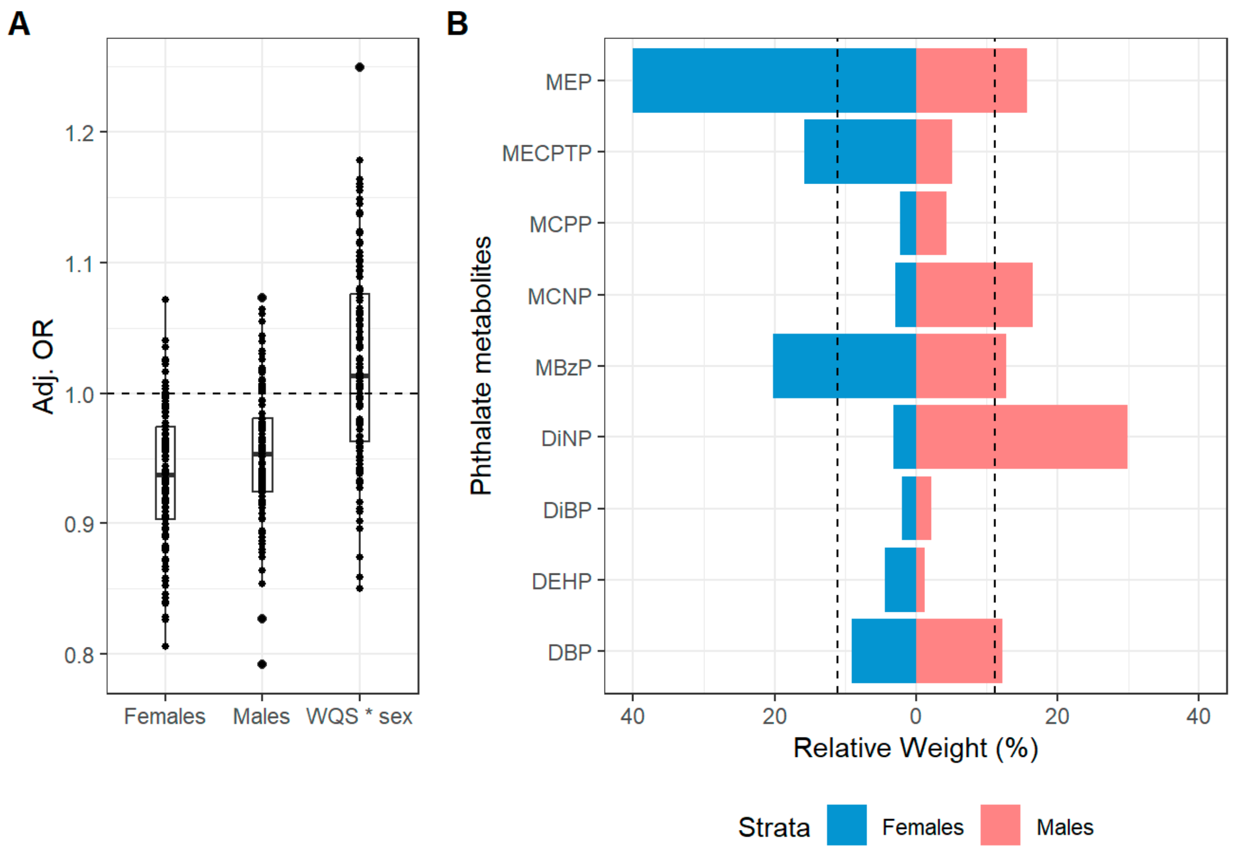

Figure S9. Mean adjusted betas (A) and sex-specific relative weights (B) from a WQS (positive constraint) linear regression model with 110 repeated holdouts between 2nd trimester phthalates mixture and ever allergic rhinitis symptoms at 6-8 years. The model was adjusted for maternal age, BMI, ETS, education, parity. (A) Illustrates the distribution of the adjusted betas across the 110 repeated holdouts where each dot represents the estimate from each holdout. (B) Illustrates the mean estimated relative weight for each chemical of the phthalate mixtures across the 110 repeated holdouts. The relative weight is the percentage of weight attributable to each chemical in the phthalate mixtures within the total weight of each strata (males and females). The dotted line represents the threshold (11.1%) for chemicals of concern. Chemicals with relative weights above this threshold in at least 50% of the repeated holdouts were considered chemicals of concern. Abbreviations: BMI = body mass index; ETS = environmental tobacco smoke; Notes: All chemicals were $\log_2$ transformed to reduce skewness in the distribution of the concentrations.

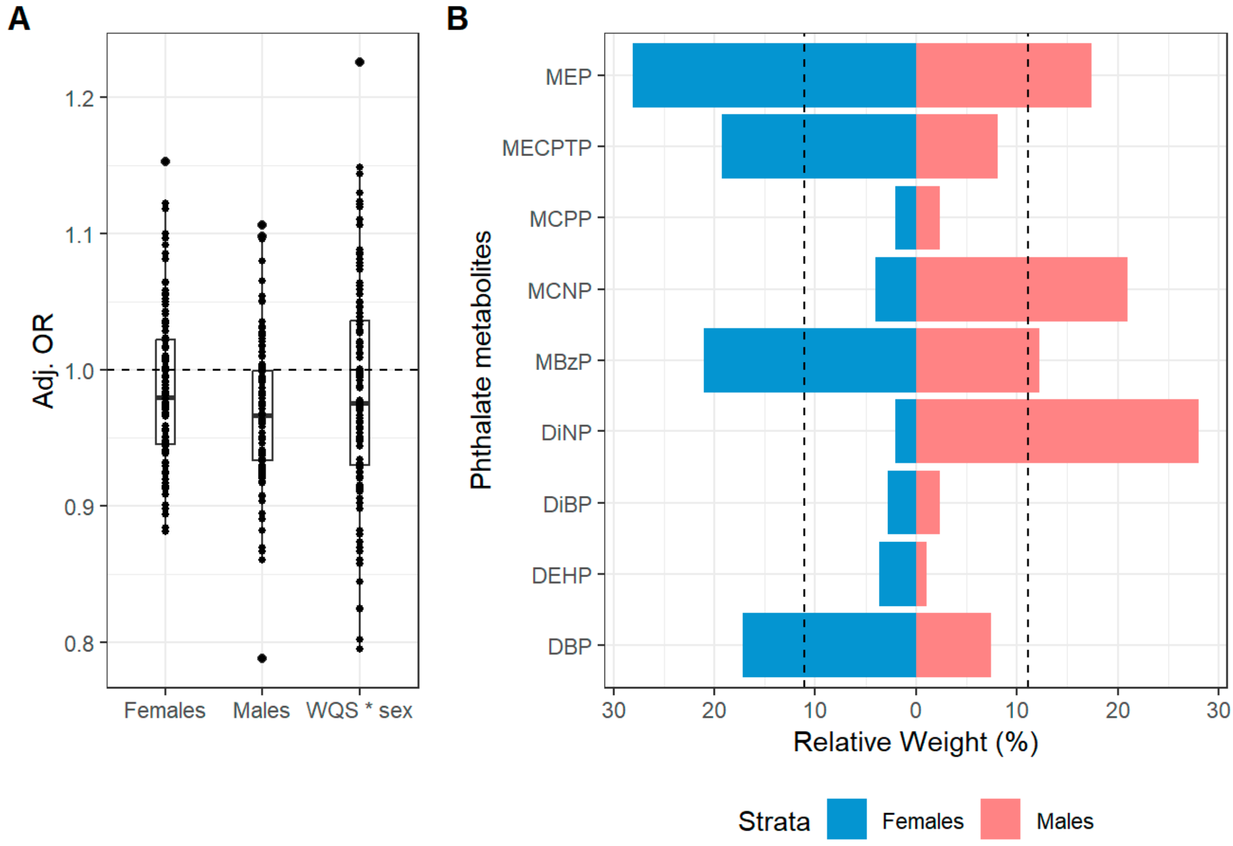

Figure S10. Mean adjusted betas (A) and sex-specific relative weights (B) from a WQS (positive constraint) linear regression model with 110 repeated holdouts between 2nd trimester phthalates mixture and current allergic rhinitis symptoms at 6-8 years. The model was adjusted for maternal age, BMI, ETS, education, parity. (A) Illustrates the distribution of the adjusted betas across the 110 repeated holdouts where each dot represents the estimate from each holdout. (B) Illustrates the mean estimated relative weight for each chemical of the phthalate mixtures across the 110 repeated holdouts. The relative weight is the percentage of weight attributable to each chemical in the phthalate mixtures within the total weight of each strata (males and females). The dotted line represents the threshold (11.1%) for chemicals of concern. Chemicals with relative weights above this threshold in at least 50% of the repeated holdouts were considered chemicals of
concern. Abbreviations: BMI = body mass index; ETS = environmental tobacco smoke; Notes: All chemicals were log_2 transformed to reduce skewness in the distribution of the concentrations.

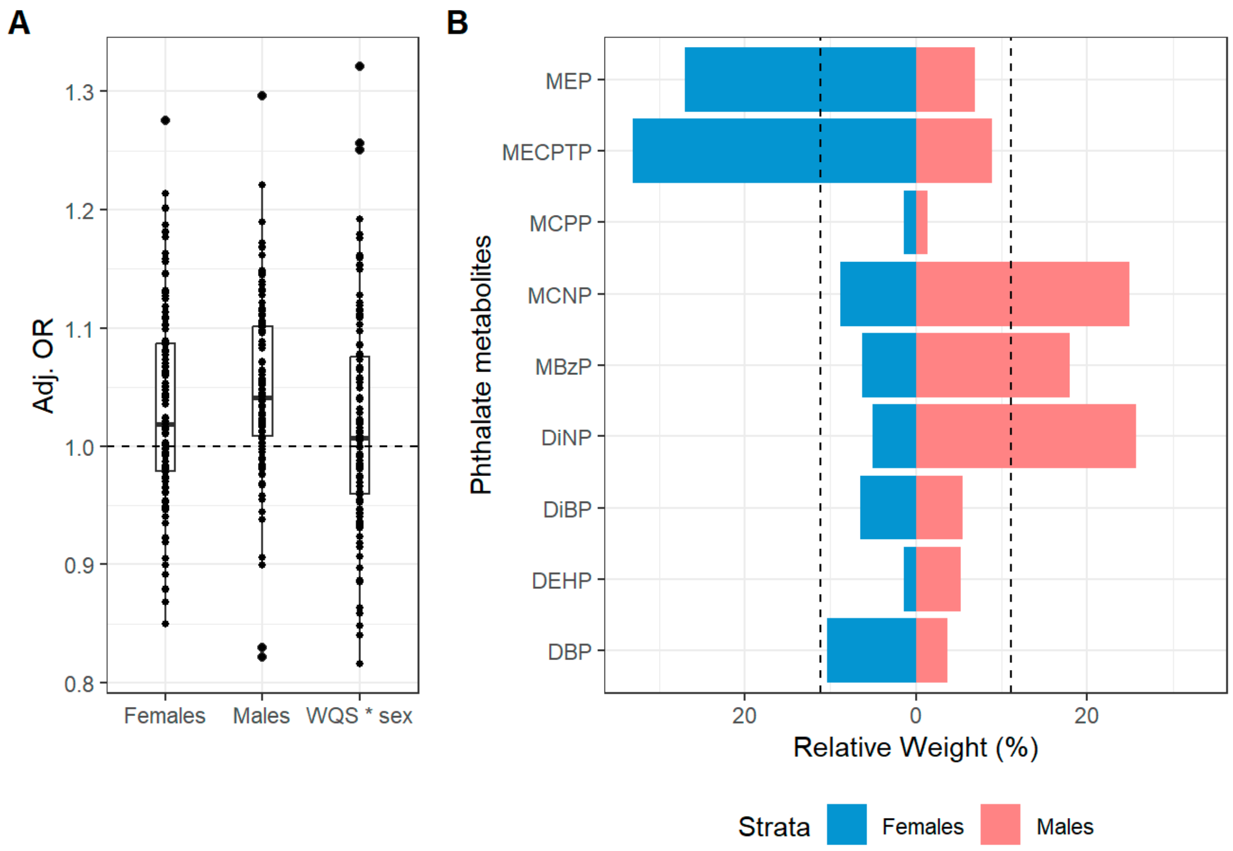

Figure S11. Mean adjusted betas (A) and sex-specific relative weights (B) from a WQS (positive constraint) linear regression model with 110 repeated holdouts between 2nd trimester phthalates mixture and current allergic rhinitis symptoms + itchy watery eyes at 6-8 years. The model was adjusted for maternal age, BMI, ETS, education, parity. (A) Illustrates the distribution of the adjusted betas across the 110 repeated holdouts where each dot represents the estimate from each holdout. (B) Illustrates the mean estimated relative weight for each chemical of the phthalate mixtures across the 110 repeated holdouts. The relative weight is the percentage of weight attributable to each chemical in the phthalate mixtures within the total weight of each strata (males and females). The dotted line represents the threshold (11.1%) for chemicals of concern. Chemicals with relative weights above this threshold in at least 50% of the repeated holdouts were considered chemicals of
concern. Abbreviations: BMI = body mass index; ETS = environmental tobacco smoke; Notes: All chemicals were $\log_2$ transformed to reduce skewness in the distribution of the concentrations.

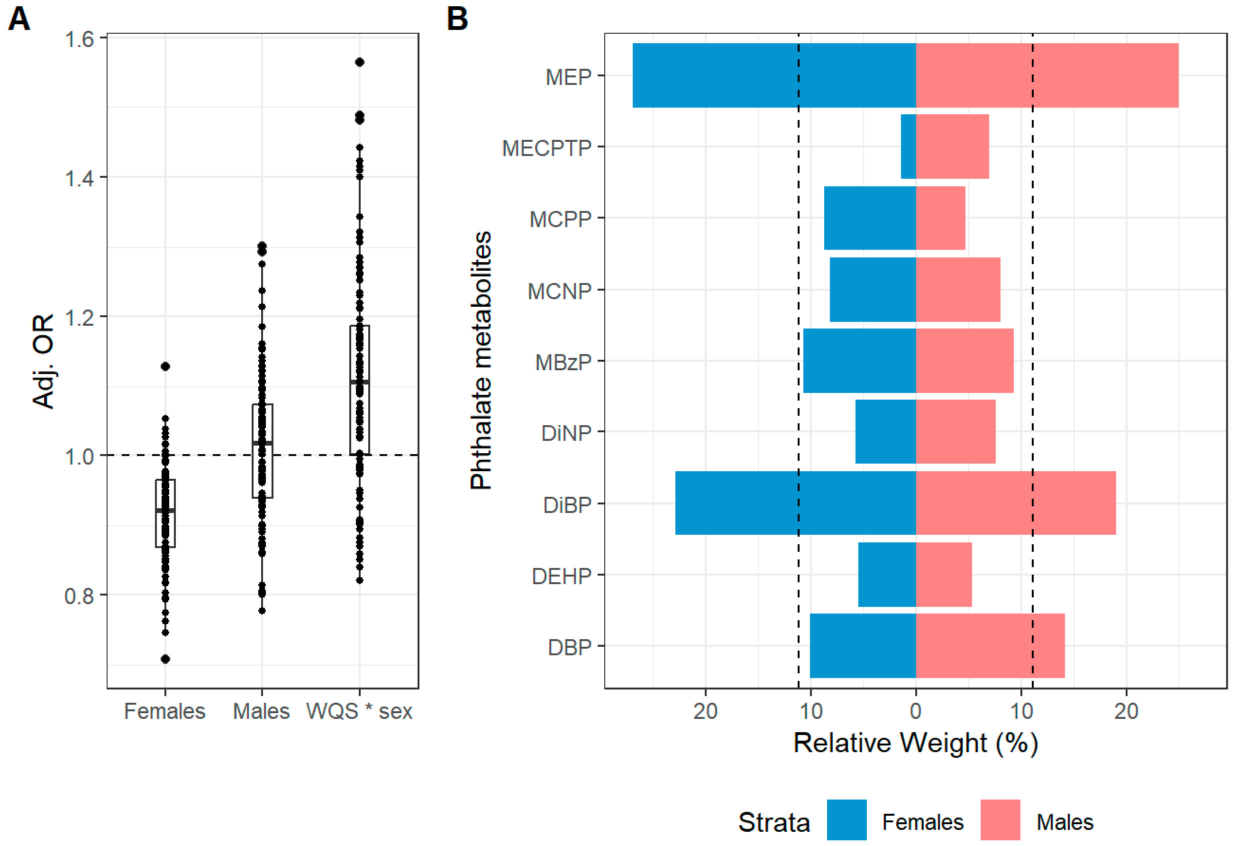

Figure S12. Mean adjusted betas (A) and sex-specific relative weights (B) from a WQS (positive constraint) linear regression model with 110 repeated holdouts between 3rd trimester phthalates mixture and ever atopic dermatitis symptoms at 4-6 years. The model was adjusted for maternal age, BMI, ETS, education, parity. (A) Illustrates the distribution of the adjusted betas across the 110 repeated holdouts where each dot represents the estimate from each holdout. (B) Illustrates the mean estimated relative weight for each chemical of the phthalate mixtures across the 110 repeated holdouts. The relative weight is the percentage of weight attributable to each chemical in the phthalate mixtures within the total weight of each strata (males and females). The dotted line represents the threshold (11.1%) for chemicals of concern. Chemicals with relative weights above this threshold in at least 50% of the repeated holdouts were considered chemicals of
concern. Abbreviations: BMI = body mass index; ETS = environmental tobacco smoke; Notes: All chemicals were $\log_2$ transformed to reduce skewness in the distribution of the concentrations.

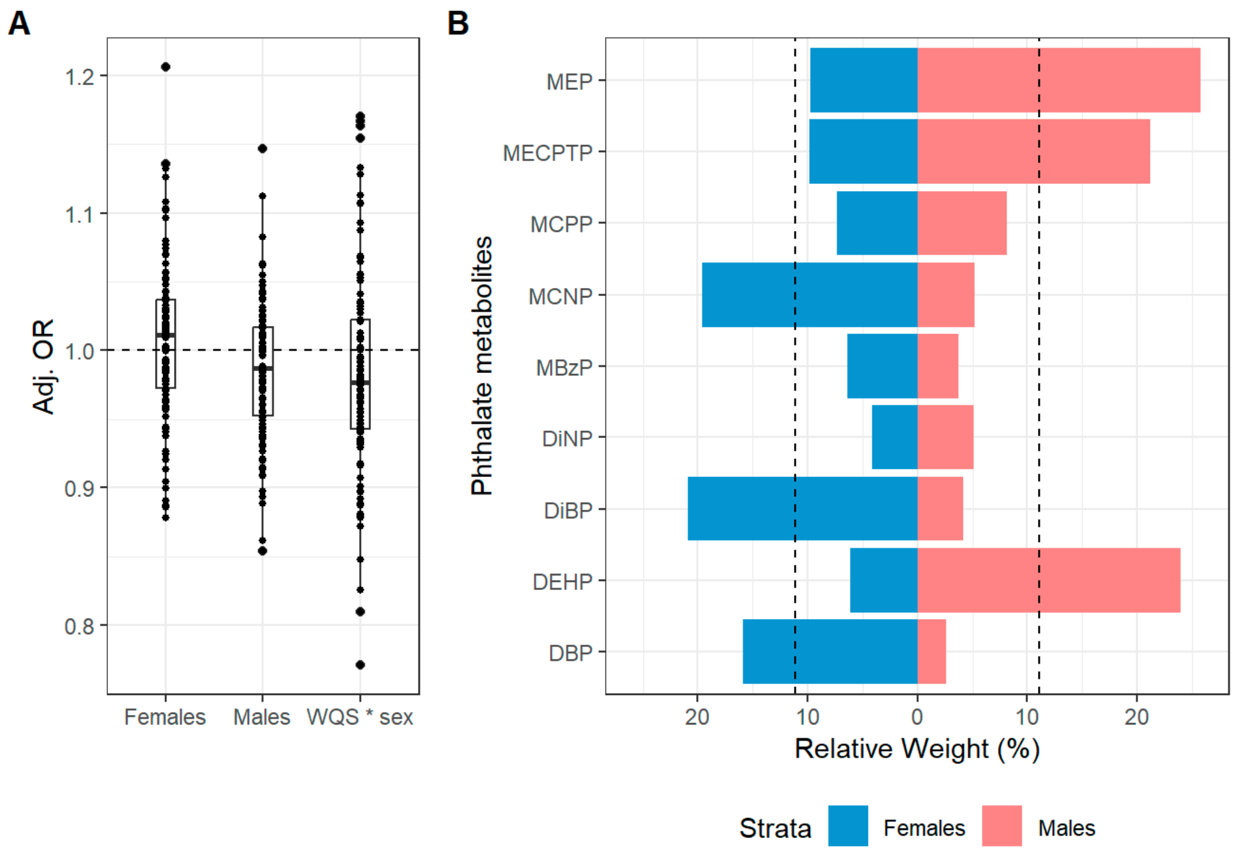

Figure S13. Mean adjusted betas (A) and sex-specific relative weights (B) from a WQS (positive constraint) linear regression model with 110 repeated holdouts between 3rd trimester phthalates mixture and ever allergic rhinitis symptoms at 4-6 years. The model was adjusted for maternal age, BMI, ETS, education, parity. (A) Illustrates the distribution of the adjusted betas across the 110 repeated holdouts where each dot represents the estimate from each holdout. (B) Illustrates the mean estimated relative weight for each chemical of the phthalate mixtures across the 110 repeated holdouts. The relative weight is the percentage of weight attributable to each chemical in the phthalate mixtures within the total weight of each strata (males and females). The dotted line represents the threshold (11.1%) for chemicals of concern. Chemicals with relative weights above this threshold in at least 50% of the repeated holdouts were considered chemicals of
concern. Abbreviations: BMI = body mass index; ETS = environmental tobacco smoke; Notes: All chemicals were $\log_2$ transformed to reduce skewness in the distribution of the concentrations.

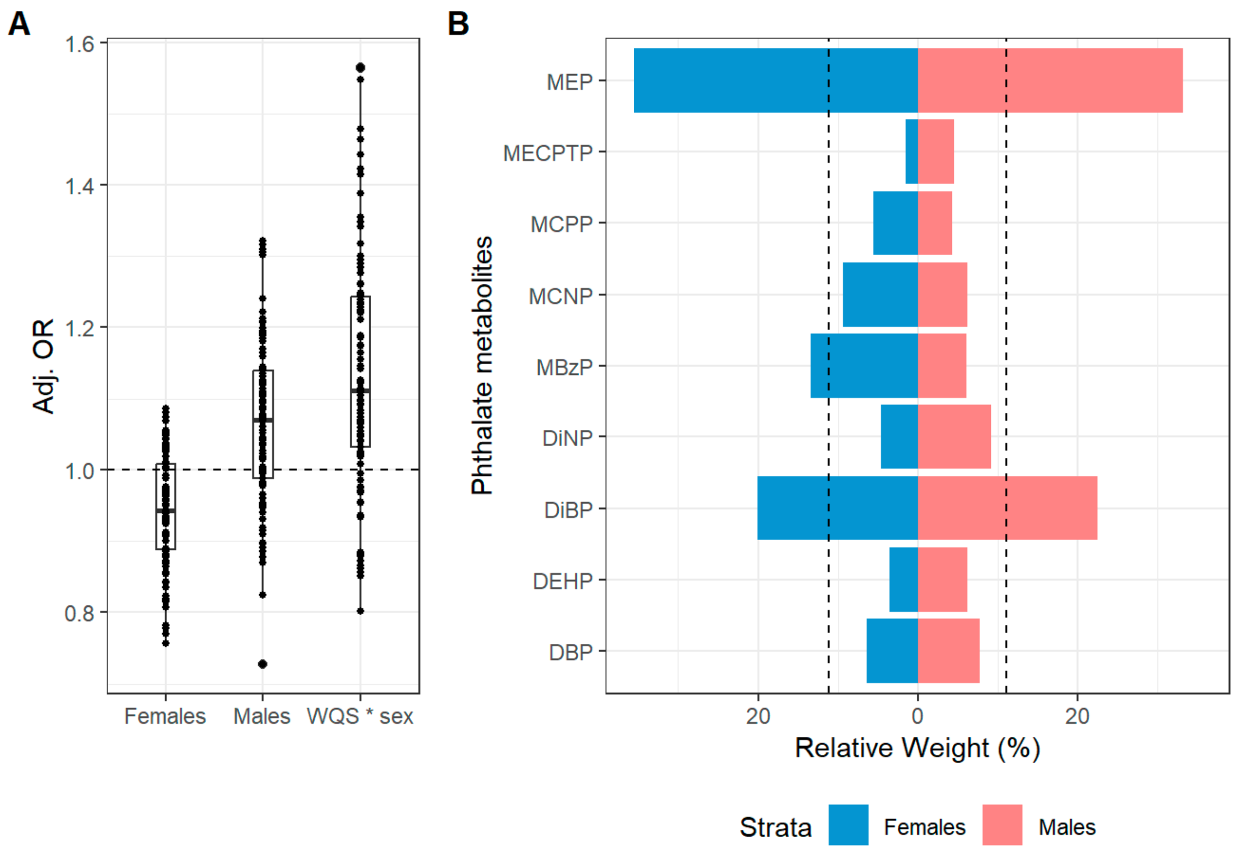

Figure S14. Mean adjusted betas (A) and sex-specific relative weights (B) from a WQS (positive constraint) linear regression model with 110 repeated holdouts between 3rd trimester phthalates mixture and current atopic dermatitis symptoms at 4-6 years. The model was adjusted for maternal age, BMI, ETS, education, parity. (A) Illustrates the distribution of the adjusted betas across the 110 repeated holdouts where each dot represents the estimate from each holdout. (B) Illustrates the mean estimated relative weight for each chemical of the phthalate mixtures across the 110 repeated holdouts. The relative weight is the percentage of weight attributable to each chemical in the phthalate mixtures within the total weight of each strata (males and females). The dotted line represents the threshold (11.1%) for chemicals of concern. Chemicals with relative weights above this threshold in at least 50% of the repeated holdouts were considered chemicals of concern. Abbreviations: BMI = body mass index; ETS = environmental tobacco smoke; Notes: All chemicals were $\log_2$ transformed to reduce skewness in the distribution of the concentrations.

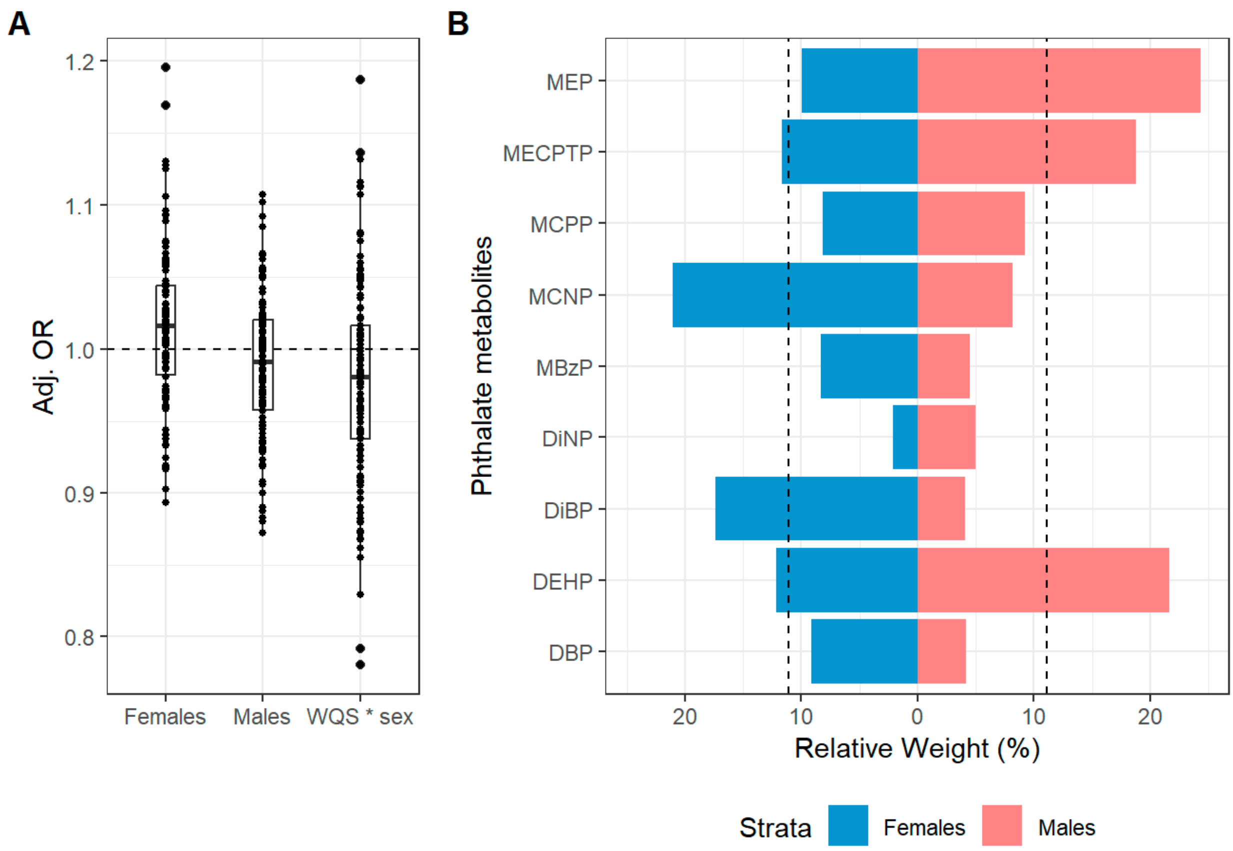

Figure S15. Mean adjusted betas (A) and sex-specific relative weights (B) from a WQS (positive constraint) linear regression model with 110 repeated holdouts between 3rd trimester phthalates mixture and current allergic rhinitis symptoms at 4-6 years. The model was adjusted for maternal age, BMI, ETS, education, parity. (A) Illustrates the distribution of the adjusted betas across the 110 repeated holdouts where each dot represents the estimate from each holdout. (B) Illustrates the mean estimated relative weight for each chemical of the phthalate mixtures across the 110 repeated holdouts. The relative weight is the percentage of weight attributable to each chemical in the phthalate mixtures within the total weight of each strata (males and females). The dotted line represents the threshold (11.1%) for chemicals of concern. Chemicals with relative weights above this threshold in at least 50% of the repeated holdouts were considered chemicals of concern. Abbreviations: BMI = body mass index; ETS = environmental tobacco smoke; Notes: All chemicals were log_2 transformed to reduce skewness in the distribution of the concentrations.

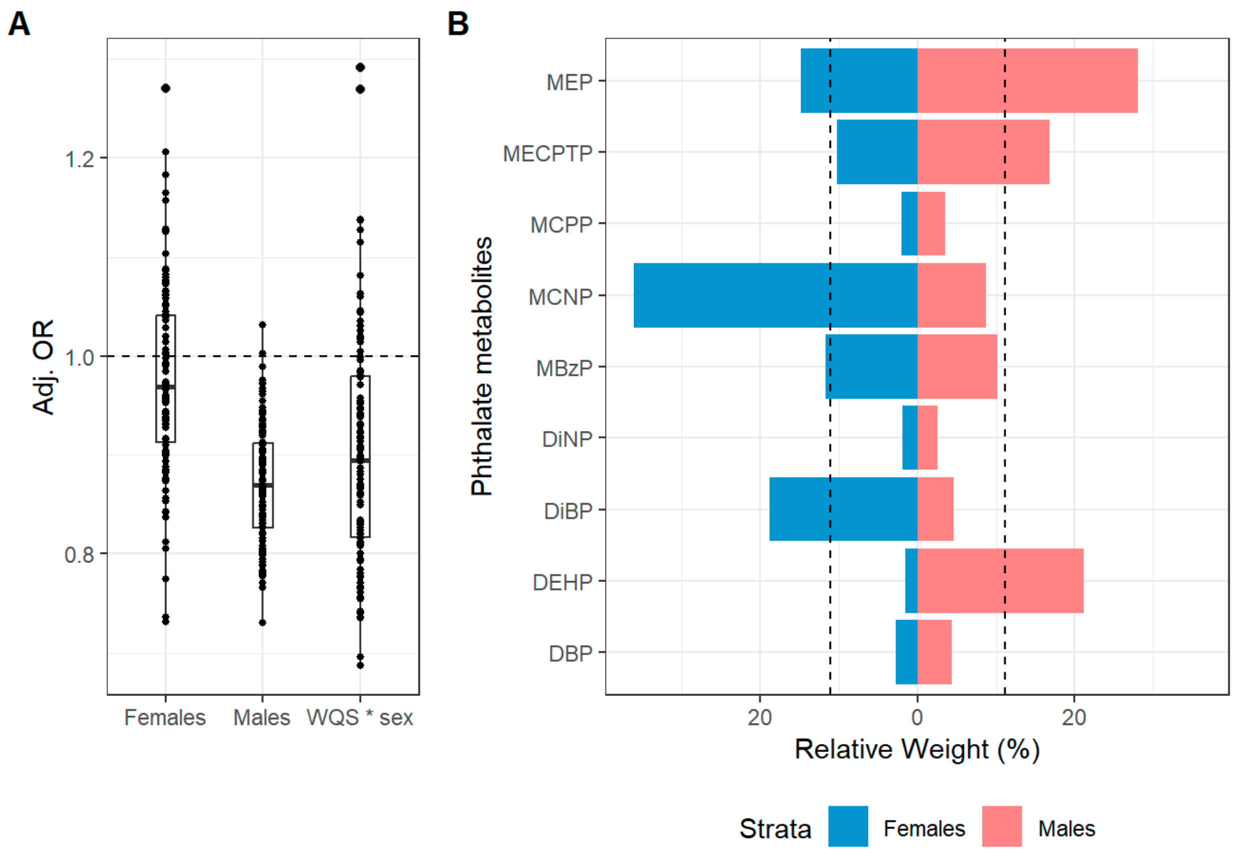

Figure S16. Mean adjusted betas (A) and sex-specific relative weights (B) from a WQS (positive constraint) linear regression model with 110 repeated holdouts between 3rd trimester phthalates mixture and current allergic rhinitis symptoms + itchy watery eyes at 4-6 years. The model was adjusted for maternal age, BMI, ETS, education, parity. (A) Illustrates the distribution of the adjusted betas across the 110 repeated holdouts where each dot represents the estimate from each holdout. (B) Illustrates the mean estimated relative weight for each chemical of the phthalate mixtures across the 110 repeated holdouts. The relative weight is the percentage of weight attributable to each chemical in the phthalate mixtures within the total weight of each strata (males and females). The dotted line represents the threshold (11.1%) for chemicals of concern. Chemicals with relative weights above this threshold in at least 50% of the repeated holdouts were considered chemicals of concern. Abbreviations: BMI = body mass index; ETS = environmental tobacco smoke; Notes: All chemicals were $\log_2$ transformed to reduce skewness in the distribution of the concentrations.

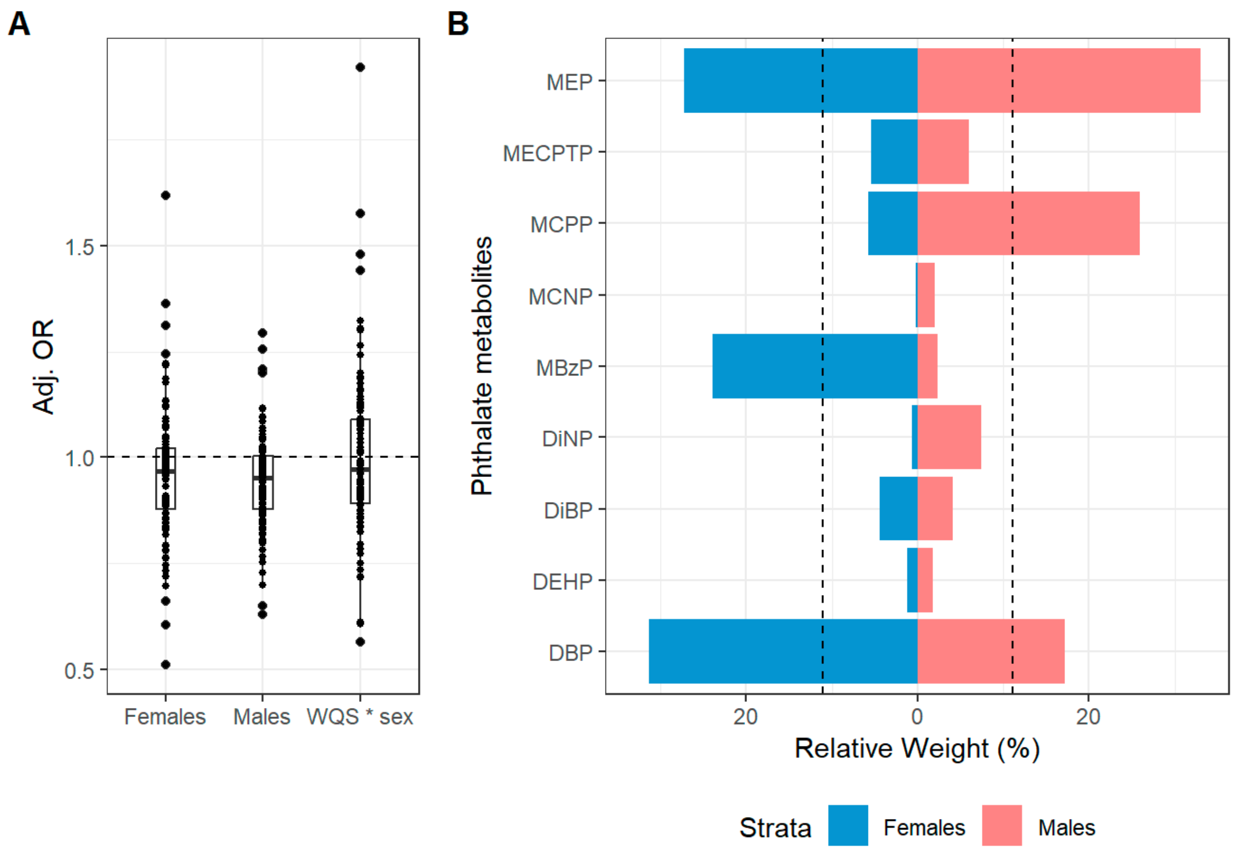

Figure S17. Mean adjusted betas (A) and sex-specific relative weights (B) from a WQS (positive constraint) linear regression model with 110 repeated holdouts between 3rd trimester phthalates mixture and ever atopic dermatitis symptoms at 6-8 years. The model was adjusted for maternal age, BMI, ETS, education, parity. (A) Illustrates the distribution of the adjusted betas across the 110 repeated holdouts where each dot represents the estimate from each holdout. (B) Illustrates the mean estimated relative weight for each chemical of the phthalate mixtures across the 110 repeated holdouts. The relative weight is the percentage of weight attributable to each chemical in the phthalate mixtures within the total weight of each strata (males and females). The dotted line represents the threshold (11.1%) for chemicals of concern. Chemicals with relative weights above this threshold in at least 50% of the repeated holdouts were considered chemicals of concern. Abbreviations: BMI = body mass index; ETS = environmental tobacco smoke; Notes: All chemicals were $\log_2$ transformed to reduce skewness in the distribution of the concentrations.

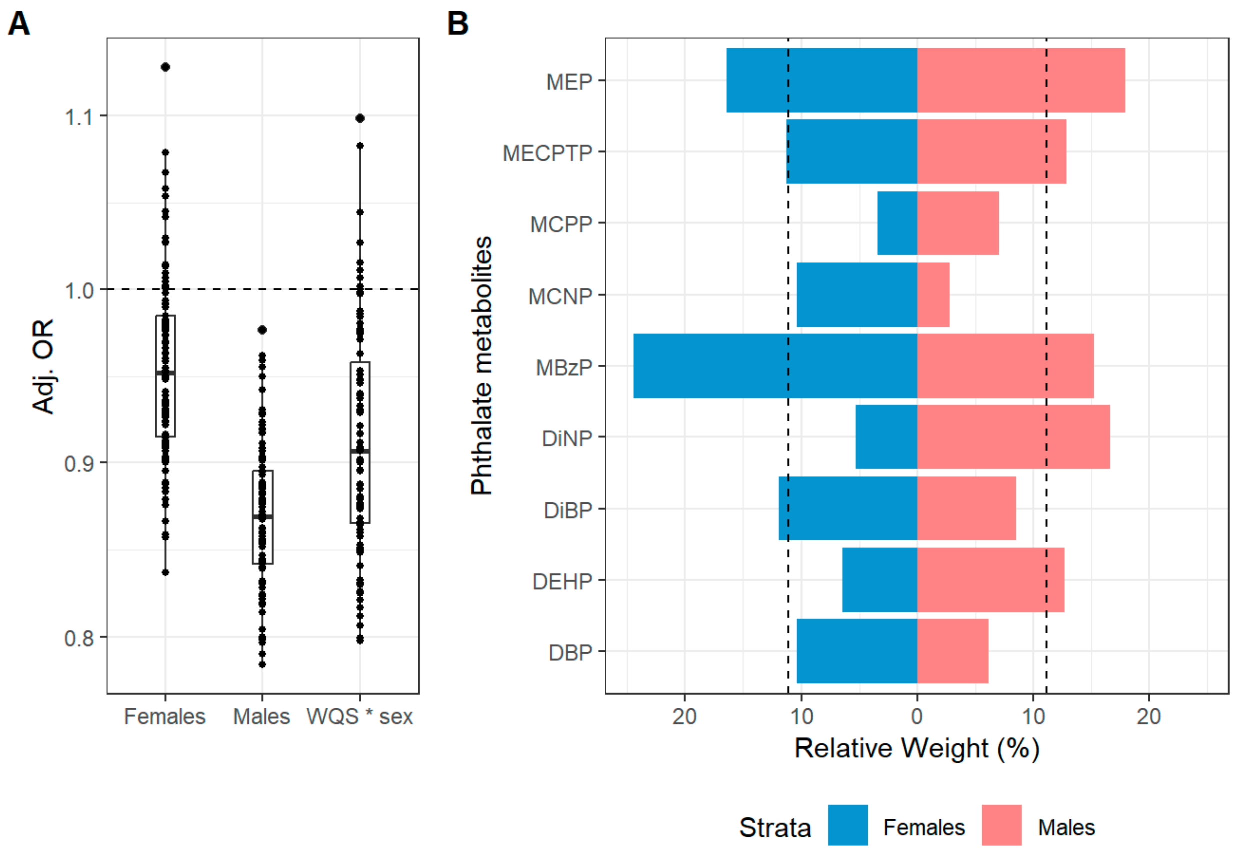

Figure S18. Mean adjusted betas (A) and sex-specific relative weights (B) from a WQS (positive constraint) linear regression model with 110 repeated holdouts between 3rd trimester phthalates mixture and ever allergic rhinitis symptoms at 6-8 years. The model was adjusted for maternal age, BMI, ETS, education, parity. (A) Illustrates the distribution of the adjusted betas across the 110 repeated holdouts where each dot represents the estimate from each holdout. (B) Illustrates the mean estimated relative weight for each chemical of the phthalate mixtures across the 110 repeated holdouts. The relative weight is the percentage of weight attributable to each chemical in the phthalate mixtures within the total weight of each strata (males and females). The dotted line represents the threshold (11.1%) for chemicals of concern. Chemicals with relative weights above this threshold in at least 50% of the repeated holdouts were considered chemicals of concern. Abbreviations: BMI = body mass index; ETS = environmental tobacco smoke; Notes: All chemicals were log_2 transformed to reduce skewness in the distribution of the concentrations.

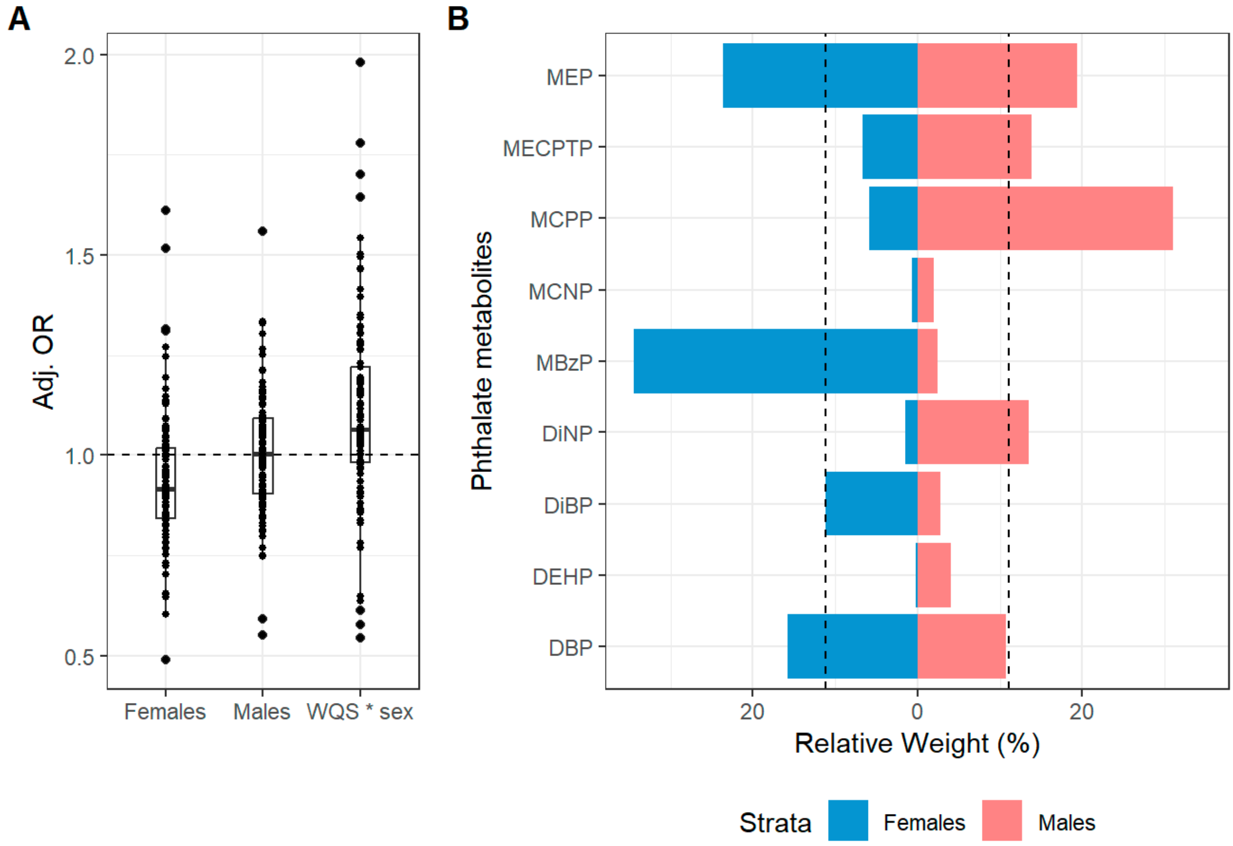

Figure S19. Mean adjusted betas (A) and sex-specific relative weights (B) from a WQS (positive constraint) linear regression model with 110 repeated holdouts between 3rd trimester phthalates mixture and current atopic dermatitis symptoms at 6-8 years. The model was adjusted for maternal age, BMI, ETS, education, parity. (A) Illustrates the distribution of the adjusted betas across the 110 repeated holdouts where each dot represents the estimate from each holdout. (B) Illustrates the mean estimated relative weight for each chemical of the phthalate mixtures across the 110 repeated holdouts. The relative weight is the percentage of weight attributable to each chemical in the phthalate mixtures within the total weight of each strata (males and females). The dotted line represents the threshold (11.1%) for chemicals of concern. Chemicals with relative weights above this threshold in at least 50% of the repeated holdouts were considered chemicals of concern. Abbreviations: BMI = body mass index; ETS = environmental tobacco smoke; Notes: All chemicals were $\log_2$ transformed to reduce skewness in the distribution of the concentrations.

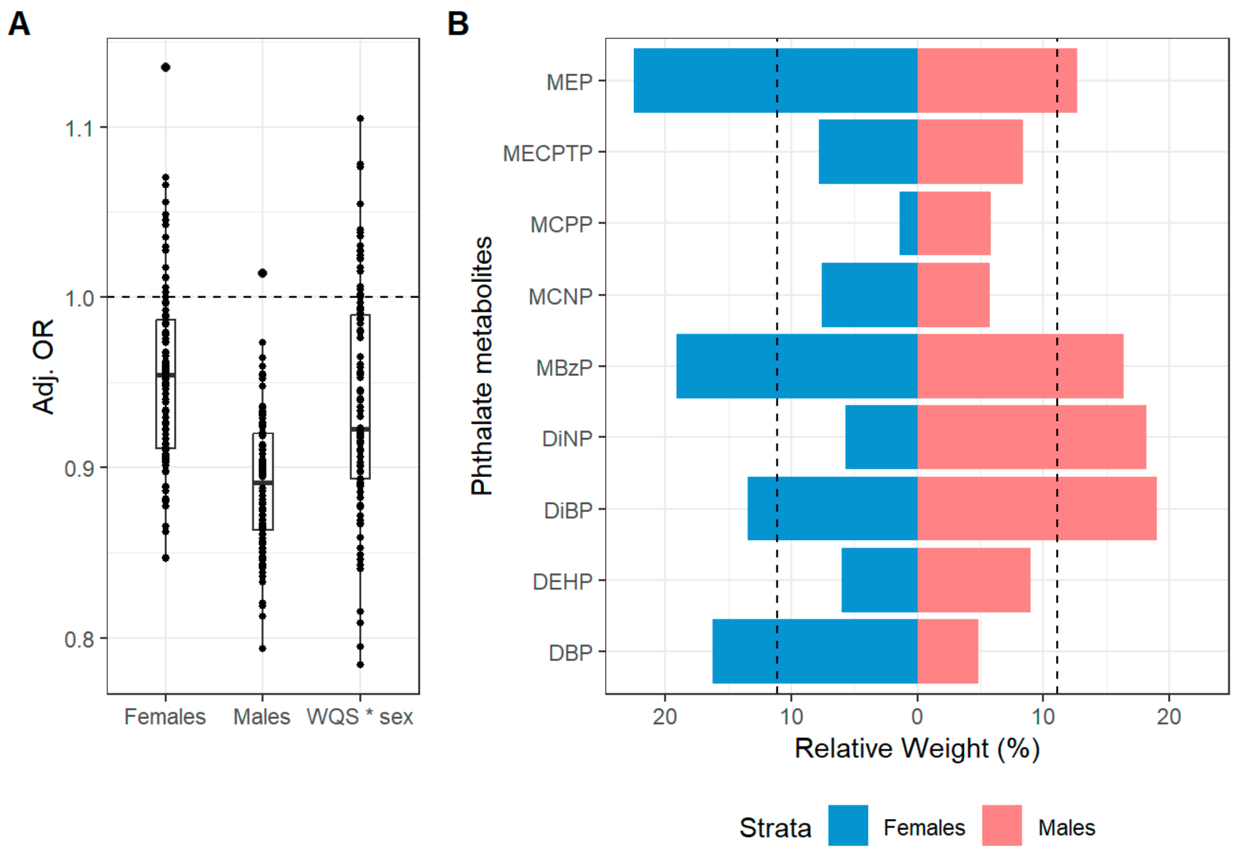

Figure S20. Mean adjusted betas (A) and sex-specific relative weights (B) from a WQS (positive constraint) linear regression model with 110 repeated holdouts between 3rd trimester phthalates mixture and current allergic rhinitis symptoms at 6-8 years. The model was adjusted for maternal age, BMI, ETS, education, parity. (A) Illustrates the distribution of the adjusted betas across the 110 repeated holdouts where each dot represents the estimate from each holdout. (B) Illustrates the mean estimated relative weight for each chemical of the phthalate mixtures across the 110 repeated holdouts. The relative weight is the percentage of weight attributable to each chemical in the phthalate mixtures within the total weight of each strata (males and females). The dotted line represents the threshold (11.1%) for chemicals of concern. Chemicals with relative weights above this threshold in at least 50% of the repeated holdouts were considered chemicals of concern. Abbreviations: BMI = body mass index; ETS = environmental tobacco smoke; Notes: All chemicals were log_2 transformed to reduce skewness in the distribution of the concentrations.

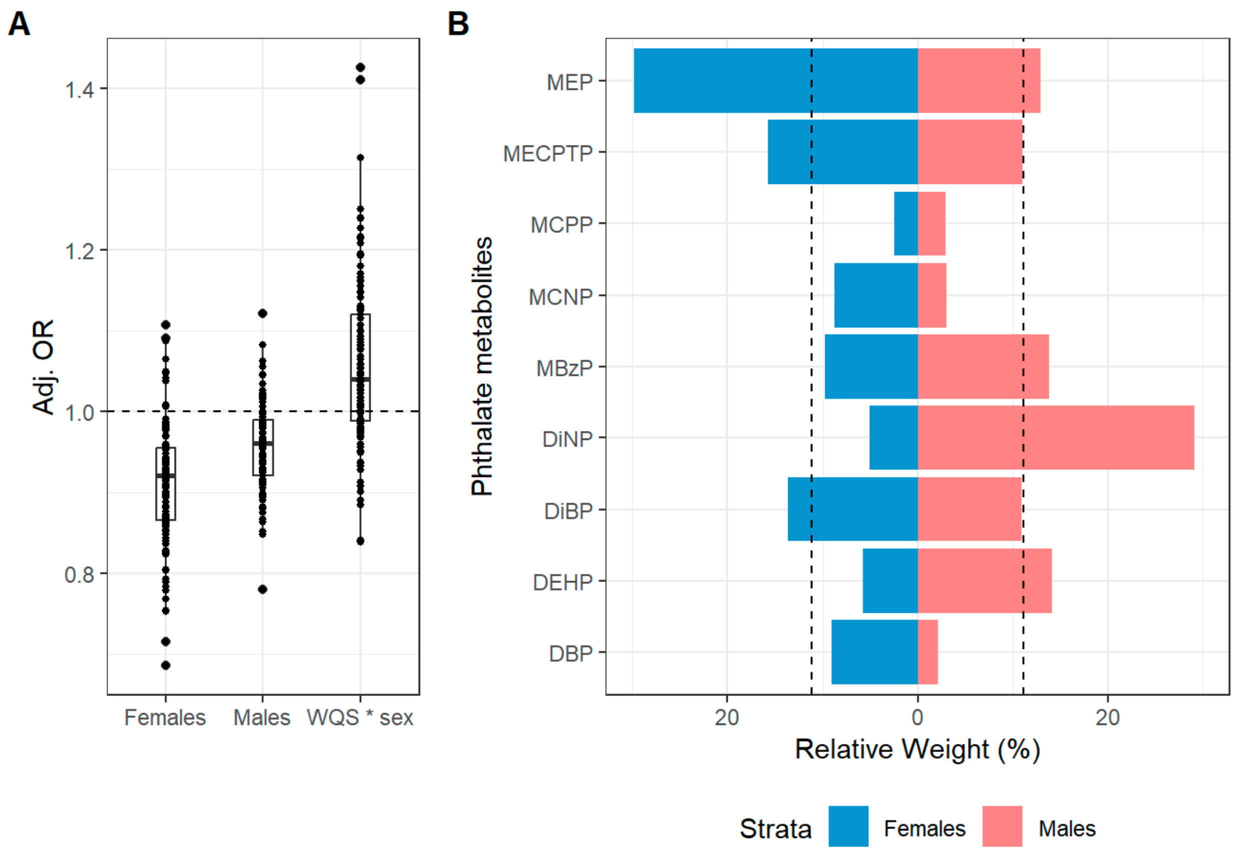

Figure S21. Mean adjusted betas (A) and sex-specific relative weights (B) from a WQS (positive constraint) linear regression model with 110 repeated holdouts between 3rd trimester phthalates mixture and current allergic rhinitis symptoms + itchy watery eyes at 6-8 years. The model was adjusted for maternal age, BMI, ETS, education, parity. (A) Illustrates the distribution of the adjusted betas across the 110 repeated holdouts where each dot represents the estimate from each holdout. (B) Illustrates the mean estimated relative weight for each chemical of the phthalate mixtures across the 110 repeated holdouts. The relative weight is the percentage of weight attributable to each chemical in the phthalate mixtures within the total weight of each strata (males and females). The dotted line represents the threshold (11.1%) for chemicals of concern. Chemicals with relative weights above this threshold in at least 50% of the repeated holdouts were considered chemicals of
concern. Abbreviations: BMI = body mass index; ETS = environmental tobacco smoke; Notes: All chemicals were $\log_2$ transformed to reduce skewness in the distribution of the concentrations.

Table S1. Mean adjusted association from WQS (positive constraint) binomial model with 110 repeated holdouts between 2nd trimester phthalates mixture and ever atopic dermatitis symptoms at 4-6 years age (N=558)

Ever atopic dermatitis symptoms [a]	Mean OR and 95% CI [b]	OR > 1 [c]
WQS (b_1)	0.88 (0.75, 1.04)	6/110
WQS*sex (b_{12})	1.17 (0.92, 1.50)	96/110
Betas for males and females		
Females (b_1) [d]	0.88 (0.75, 1.04)	6/110
Males (b_2) [d]	1.04 (0.86, 1.25)	65/110

Abbreviations: WQS, weighted quantile sum; OR, Odds Ratio, CI, confidence interval; BMI, body mass index; ETS, environmental tobacco smoke.

Notes: All chemicals were log_2 transformed to reduce skewness in the distribution of the concentrations.

*[a] The association is derived from a stratified WQS linear regression model allowing for sex-specific weights and including an interaction term WQS*sex. The model was run with 110 repeated holdouts using 40% of the data as training and 60% as validation set. The models were adjusted for maternal age, pre-pregnancy BMI, ETS exposure during pregnancy, education at enrollment, and parity.*

[b] The mean beta over the 110 repeated holdouts is presented with its 95% confidence interval.

[c] The number of adjusted betas from the 110 repeated holdouts that were positive.

*[d] Adjusted beta for females (b_1) is the beta for the WQS index for the reference group (females = 0), and the adjusted beta for males (b_2), which is the comparison group (males = 1) is calculated based on the sum of the beta for the WQS*sex interaction term and the beta for the WQS index for the reference group ($b_2 = b_1 + b_{12}$).*

Table S2. Mean adjusted association from WQS (positive constraint) binomial model with 110 repeated holdouts between 2nd trimester phthalates mixture and ever allergic rhinitis symptoms at 4-6 years age (N=558)

Ever allergic rhinitis symptoms [a]	Mean OR and 95% CI [b]	OR > 1 [c]
WQS (b_1)	1.04 (0.95, 1.14)	87/110
WQS*sex (b_{12})	0.94 (0.82, 1.07)	22/110
Betas for males and females		

Females (b_1) [d]	1.04 (0.95, 1.14)	87/110
Males (b_2) [d]	0.97 (0.88, 1.07)	32/110

Abbreviations: WQS, weighted quantile sum; OR, Odds Ratio, CI, confidence interval; BMI, body mass index; ETS, environmental tobacco smoke.

Notes: All chemicals were log_2 transformed to reduce skewness in the distribution of the concentrations.

*[a] The association is derived from a stratified WQS linear regression model allowing for sex-specific weights and including an interaction term WQS*sex. The model was run with 110 repeated holdouts using 40% of the data as training and 60% as validation set. The models were adjusted for maternal age, pre-pregnancy BMI, ETS exposure during pregnancy, education at enrollment, and parity.*

[b] The mean beta over the 110 repeated holdouts is presented with its 95% confidence interval.

[c] The number of adjusted betas from the 110 repeated holdouts that were positive.

*[d] Adjusted beta for females (b_1) is the beta for the WQS index for the reference group (females = 0), and the adjusted beta for males (b_2), which is the comparison group (males = 1) is calculated based on the sum of the beta for the WQS*sex interaction term and the beta for the WQS index for the reference group ($b_2 = b_1 + b_{12}$).*

Table S3. Mean adjusted association from WQS (positive constraint) binomial model with 110 repeated holdouts between 2nd trimester phthalates mixture and current allergic rhinitis symptoms at 4-6 years age (N=558)

Current allergic rhinitis symptoms [a]	Mean OR and 95% CI [b]	OR > 1 [c]
WQS (b_1)	1.06 (0.97, 1.15)	98/110
WQS*sex (b_{12})	0.92 (0.82, 1.04)	14/110
Betas for males and females		
Females (b_1) [d]	1.06 (0.97, 1.15)	98/110
Males (b_2) [d]	0.97 (0.89, 1.06)	30/110

Abbreviations: WQS, weighted quantile sum; OR, Odds Ratio, CI, confidence interval; BMI, body mass index; ETS, environmental tobacco smoke.

Notes: All chemicals were log_2 transformed to reduce skewness in the distribution of the concentrations.

*[a] The association is derived from a stratified WQS linear regression model allowing for sex-specific weights and including an interaction term WQS*sex. The model was run with 110 repeated holdouts using 40% of the data as training and 60% as validation set. The models were adjusted for maternal age, pre-pregnancy BMI, ETS exposure during pregnancy, education at enrollment, and parity.*

[b] The mean beta over the 110 repeated holdouts is presented with its 95% confidence interval.

[c] The number of adjusted betas from the 110 repeated holdouts that were positive.

*[d] Adjusted beta for females (b_1) is the beta for the WQS index for the reference group (females = 0), and the adjusted beta for males (b_2), which is the comparison group (males = 1) is calculated based on the sum of the beta for the WQS*sex interaction term and the beta for the WQS index for the reference group ($b_2 = b_1 + b_{12}$).*

Table S4. Mean adjusted association from WQS (positive constraint) binomial model with 110 repeated holdouts between 2nd trimester phthalates mixture and current allergic rhinitis symptoms + itchy watery eyes at 4-6 years age (N=558)

Current allergic rhinitis symptoms + itchy watery eyes [a]	Mean OR and 95% CI [b]	OR > 1 [c]
WQS (b_1)	1.04 (0.90, 1.21)	76/110
WQS*sex (b_{12})	0.86 (0.70, 1.06)	7/110
Betas for males and females		
Females (b_1) [d]	1.04 (0.90, 1.21)	76/110
Males (b_2) [d]	0.90 (0.78, 1.04)	10/110

Abbreviations: WQS (weighted quantile sum), OR (Odds Ratio), CI (confidence interval), BMI (body mass index), ETS (environmental tobacco smoke).
All compounds underwent log2 transformation to decrease skewness in the concentration distribution.
*a) The association is based on a stratified Weighted Quantile Sum (WQS) linear regression model that incorporates sex-specific weights and features an interaction term WQS*sex. The model was executed using 110 repeated holdouts, utilizing 40% of the data for training and 60% for validation. The models were modified for mother age, pre-pregnancy BMI, exposure to environmental tobacco smoke during pregnancy, educational attainment at enrollment, and parity.*
b) The average beta throughout the 110 repeated holdouts is displayed along with its 95% confidence interval.
c) The quantity of corrected betas from the 110 repeated holdouts that were positive.
*d) The adjusted beta for females (b1) represents the beta for the WQS index within the reference group (females = 0), while the adjusted beta for males (b2), the comparison group (males = 1), is determined by the sum of the beta for the WQS*sex interaction term and the beta for the WQS index for the reference group (b2 = b1 + b12).*

Table S5. Mean adjusted association from WQS (positive constraint) binomial model with 110 repeated holdouts between 2nd trimester phthalates mixture and ever atopic dermatitis symptoms at 6-8 years (N=558)

Ever atopic dermatitis symptoms [a]	Mean OR and 95% CI [b]	OR > 1 [c]
WQS (b_1)	0.89 (0.72, 1.11)	18/110
WQS*sex (b_{12})	1.12 (0.84, 1.49)	81/110
Betas for males and females		
Females (b_1) [d]	0.89 (0.72, 1.11)	18/110
Males (b_2) [d]	1.00 (0.83, 1.20)	58/110

Abbreviations: WQS, weighted quantile sum; OR, Odds Ratio, CI, confidence interval; BMI, body mass index; ETS, environmental tobacco smoke.

Notes: All chemicals were log_2 transformed to reduce skewness in the distribution of the concentrations.

*[a] The association is derived from a stratified WQS linear regression model allowing for sex-specific weights and including an interaction term WQS*sex. The model was run with 110 repeated holdouts using 40% of the data as training and 60% as validation set. The models were adjusted for maternal age, pre-pregnancy BMI, ETS exposure during pregnancy, education at enrollment, and parity.*

[b] The mean beta over the 110 repeated holdouts is presented with its 95% confidence interval.

[c] The number of adjusted betas from the 110 repeated holdouts that were positive.

*[d] Adjusted beta for females (b_1) is the beta for the WQS index for the reference group (females = 0), and the adjusted beta for males (b_2), which is the comparison group (males = 1) is calculated based on the sum of the beta for the WQS*sex interaction term and the beta for the WQS index for the reference group ($b_2 = b_1 + b_{12}$).*

Table S6. Mean adjusted association from WQS (positive constraint) binomial model with 110 repeated holdouts between 2nd trimester phthalates mixture and ever allergic rhinitis symptoms at 6-8 years age (N=558)

Ever allergic rhinitis symptoms [a]	Mean OR and 95% CI [b]	OR > 1 [c]
WQS (b_1)	0.93 (0.83, 1.05)	12/110
WQS*sex (b_{12})	1.02 (0.88, 1.18)	64/110
Betas for males and females		

Females (b_1) [d]	0.93 (0.83, 1.05)	12/110
Males (b_2) [d]	0.95 (0.86, 1.06)	22/110

Abbreviations: WQS, weighted quantile sum; OR, Odds Ratio, CI, confidence interval; BMI, body mass index; ETS, environmental tobacco smoke.

Notes: All chemicals were $\log_2$ transformed to reduce skewness in the distribution of the concentrations.

*[a] The association is derived from a stratified WQS linear regression model allowing for sex-specific weights and including an interaction term WQS*sex. The model was run with 110 repeated holdouts using 40% of the data as training and 60% as validation set. The models were adjusted for maternal age, pre-pregnancy BMI, ETS exposure during pregnancy, education at enrollment, and parity.*

[b] The mean beta over the 110 repeated holdouts is presented with its 95% confidence interval.

[c] The number of adjusted betas from the 110 repeated holdouts that were positive.

Table S7. Mean adjusted association from WQS (positive constraint) binomial model with 110 repeated holdouts between 2nd trimester phthalates mixture and current allergic rhinitis symptoms at 6-8 years age (N=558)

Current allergic rhinitis symptoms [a]	Mean OR and 95% CI [b]	OR > 1 [c]
WQS (b_1)	0.98 (0.88, 1.10)	40/110
WQS*sex (b_{12})	0.98 (0.84, 1.15)	41/110
Betas for males and females		
Females (b_1) [d]	0.98 (0.88, 1.10)	40/110
Males (b_2) [d]	0.97 (0.87, 1.08)	26/110

Abbreviations: WQS, weighted quantile sum; OR, Odds Ratio, CI, confidence interval; BMI, body mass index; ETS, environmental tobacco smoke.

Notes: All chemicals were $\log_2$ transformed to reduce skewness in the distribution of the concentrations.

*[a] The association is derived from a stratified WQS linear regression model allowing for sex-specific weights and including an interaction term WQS*sex. The model was run with 110 repeated holdouts using 40% of the data as training and 60% as validation set. The models were adjusted for maternal age, pre-pregnancy BMI, ETS exposure during pregnancy, education at enrollment, and parity.*

[b] The mean beta over the 110 repeated holdouts is presented with its 95% confidence interval.

[c] The number of adjusted betas from the 110 repeated holdouts that were positive

Table S8. Mean adjusted association from WQS (positive constraint) binomial model with 110 repeated holdouts between 2nd trimester phthalates mixture and Current allergic rhinitis symptoms + itchy watery eyes at 6-8 years age (N=558)

Current allergic rhinitis symptoms + itchy watery eyes [a]	Mean OR and 95% CI [b]	OR > 1 [c]
WQS (b_1)	1.03 (0.90, 1.21)	67/110
WQS*sex (b_{12})	1.02 (0.85, 1.21)	58/110
Betas for males and females		
Females (b_1) [d]	1.03 (0.90, 1.21)	67/110
Males (b_2) [d]	1.05 (0.91, 1.20)	10/110

Abbreviations: WQS (weighted quantile sum), OR (Odds Ratio), CI (confidence interval), BMI (body mass index), ETS (environmental tobacco smoke).
All compounds underwent log2 transformation to decrease skewness in the concentration distribution.
*a) The association is based on a stratified Weighted Quantile Sum (WQS) linear regression model that incorporates sex-specific weights and features an interaction term WQS*sex. The model was executed using 110 repeated holdouts, utilizing 40% of the data for training and 60% for validation. The models were modified for mother age, pre-pregnancy BMI, exposure to environmental tobacco smoke during pregnancy, educational attainment at enrollment, and parity.*
b) The average beta throughout the 110 repeated holdouts is displayed along with its 95% confidence interval.
c) The quantity of corrected betas from the 110 repeated holdouts that were positive.
*d) The adjusted beta for females (b1) represents the beta for the WQS index within the reference group (females = 0), while the adjusted beta for males (b2), the comparison group (males = 1), is determined by the sum of the beta for the WQS*sex interaction term and the beta for the WQS index for the reference group (b2 = b1 + b12).*

Table S9. Mean adjusted association from WQS (positive constraint) binomial model with 110 repeated holdouts between 3rd trimester phthalates mixture and ever atopic dermatitis symptoms at 4-6 years age (N= 489)

Ever atopic dermatitis symptoms [a]	Mean OR and 95% CI [b]	OR > 1 [c]
WQS (b_1)	0.91 (0.79, 1.06)	10/110
WQS*sex (b_{12})	1.10 (0.84, 1.44)	81/110
Betas for males and females		

Females (b_1) [d]	0.91 (0.79, 1.06)	10/110
Males (b_2) [d]	1.00 (0.82, 1.24)	59/110

Abbreviations: WQS, weighted quantile sum; OR, Odds Ratio, CI, confidence interval; BMI, body mass index; ETS, environmental tobacco smoke.

Notes: All chemicals were log_2 transformed to reduce skewness in the distribution of the concentrations.

*[a] The association is derived from a stratified WQS linear regression model allowing for sex-specific weights and including an interaction term WQS*sex. The model was run with 110 repeated holdouts using 40% of the data as training and 60% as validation set. The models were adjusted for maternal age, pre-pregnancy BMI, ETS exposure during pregnancy, education at enrollment, and parity.*

[b] The mean beta over the 110 repeated holdouts is presented with its 95% confidence interval.

[c] The number of adjusted betas from the 110 repeated holdouts that were positive

Table S10. Mean adjusted association from WQS (positive constraint) binomial model with 110 repeated holdouts between 3rd trimester phthalates mixture and ever allergic rhinitis symptoms at 4-6 years age (N= 489)

Ever allergic rhinitis symptoms [a]	Mean OR and 95% CI [b]	OR > 1 [c]
WQS (b_1)	1.00 (0.90, 1.21)	64/110
WQS*sex (b_{12})	0.98 (0.84, 1.14)	39/110
Betas for males and females		
Females (b_1) [d]	1.00 (0.90, 1.21)	64/110
Males (b_2) [d]	0.98 (0.89, 1.09)	46/110

Abbreviations: WQS, weighted quantile sum; OR, Odds Ratio, CI, confidence interval; BMI, body mass index; ETS, environmental tobacco smoke.

Notes: All chemicals were log_2 transformed to reduce skewness in the distribution of the concentrations.

*[a] The association is derived from a stratified WQS linear regression model allowing for sex-specific weights and including an interaction term WQS*sex. The model was run with 110 repeated holdouts using 40% of the data as training and 60% as validation set. The models were adjusted for maternal age, pre-pregnancy BMI, ETS exposure during pregnancy, education at enrollment, and parity.*

[b] The mean beta over the 110 repeated holdouts is presented with its 95% confidence interval.

[c] *The number of adjusted betas from the 110 repeated holdouts that were positive*

Table S11. Mean adjusted association from WQS (positive constraint) binomial model with 110 repeated holdouts between 3rd trimester phthalates mixture and current atopic dermatitis symptoms at 4-6 years age (N= 489)

Current atopic dermatitis symptoms[a]	Mean OR and 95% CI [b]	OR > 1 [c]
WQS (b_1)	0.94 (0.80, 1.11)	31/110
WQS*sex (b_{12})	1.12 (0.84, 1.50)	87/110
Betas for males and females		
Females (b_1) [d]	0.94 (0.80, 1.11)	31/110
Males (b_2) [d]	1.06 (0.86, 1.31)	78/110

Abbreviations: WQS, weighted quantile sum; OR, Odds Ratio, CI, confidence interval; BMI, body mass index; ETS, environmental tobacco smoke.

Notes: All chemicals were log_2 transformed to reduce skewness in the distribution of the concentrations.

[a] *The association is derived from a stratified WQS linear regression model allowing for sex-specific weights and including an interaction term WQS*sex. The model was run with 110 repeated holdouts using 40% of the data as training and 60% as validation set. The models were adjusted for maternal age, pre-pregnancy BMI, ETS exposure during pregnancy, education at enrollment, and parity.*

[b] *The mean beta over the 110 repeated holdouts is presented with its 95% confidence interval.*

[c] *The number of adjusted betas from the 110 repeated holdouts that were positive*

Table S12. Mean adjusted association from WQS (positive constraint) binomial model with 110 repeated holdouts between 3rd trimester phthalates mixture and current allergic rhinitis symptoms at 4-6 years age (N= 489)

Current allergic rhinitis symptoms [a]	Mean OR and 95% CI [b]	OR > 1 [c]
WQS (b_1)	1.01 (0.91, 1.12)	67/110
WQS*sex (b_{12})	0.97 (0.84, 1.13)	38/110
Betas for males and females		

Females (b_1) [d]	1.01 (0.91, 1.12)	67/110
Males (b_2) [d]	0.99 (0.89, 1.09)	48/110

Abbreviations: WQS, weighted quantile sum; OR, Odds Ratio, CI, confidence interval; BMI, body mass index; ETS, environmental tobacco smoke.

Notes: All chemicals were log_2 transformed to reduce skewness in the distribution of the concentrations.

*[a] The association is derived from a stratified WQS linear regression model allowing for sex-specific weights and including an interaction term WQS*sex. The model was run with 110 repeated holdouts using 40% of the data as training and 60% as validation set. The models were adjusted for maternal age, pre-pregnancy BMI, ETS exposure during pregnancy, education at enrollment, and parity.*

[b] The mean beta over the 110 repeated holdouts is presented with its 95% confidence interval.

[c] The number of adjusted betas from the 110 repeated holdouts that were positive

Table S13. Mean adjusted association from WQS (positive constraint) binomial model with 110 repeated holdouts between 3rd trimester phthalates mixture and Current allergic rhinitis symptoms + itchy watery eyes at 4-6 years age (N=489)

Current allergic rhinitis symptoms + itchy watery eyes [a]	Mean OR and 95% CI [b]	OR > 1 [c]
WQS (b_1)	0.97 (0.80, 1.20)	42/110
WQS*sex (b_{12})	0.90 (0.70, 1.10)	19/110
Betas for males and females		
Females (b_1) [d]	0.97 (0.80, 1.20)	42/110
Males (b_2) [d]	0.87 (0.76, 1.00)	2/110

Abbreviations: WQS (weighted quantile sum), OR (Odds Ratio), CI (confidence interval), BMI (body mass index), ETS (environmental tobacco smoke).
All compounds underwent log2 transformation to decrease skewness in the concentration distribution.
*a) The association is based on a stratified Weighted Quantile Sum (WQS) linear regression model that incorporates sex-specific weights and features an interaction term WQS*sex. The model was executed using 110 repeated holdouts, utilizing 40% of the data for training and 60% for validation. The models were modified for mother age, pre-pregnancy BMI, exposure to environmental tobacco smoke during pregnancy, educational attainment at enrollment, and parity.*
b) The average beta throughout the 110 repeated holdouts is displayed along with its 95% confidence interval.
c) The quantity of corrected betas from the 110 repeated holdouts that were positive.

*d) The adjusted beta for females (b1) represents the beta for the WQS index within the reference group (females = 0), while the adjusted beta for males (b2), the comparison group (males = 1), is determined by the sum of the beta for the WQS*sex interaction term and the beta for the WQS index for the reference group (b2 = b1 + b12).*

Table S14. Mean adjusted association from WQS (positive constraint) binomial model with 110 repeated holdouts between 3rd trimester phthalates mixture and ever atopic dermatitis symptoms at 6-8 years age (N= 489)

Ever atopic dermatitis symptoms [a]	Mean OR and 95% CI [b]	OR > 1 [c]
WQS (b_1)	0.95 (0.69, 1.29)	35/110
WQS*sex (b_{12})	0.99 (0.68, 1.42)	46/110
Betas for males and females		
Females (b_1) [d]	0.95 (0.69, 1.29)	35/110
Males (b_2) [d]	0.94 (0.74, 1.19)	28/110

Abbreviations: WQS, weighted quantile sum; OR, Odds Ratio, CI, confidence interval; BMI, body mass index; ETS, environmental tobacco smoke.

Notes: All chemicals were log_2 transformed to reduce skewness in the distribution of the concentrations.

*[a] The association is derived from a stratified WQS linear regression model allowing for sex-specific weights and including an interaction term WQS*sex. The model was run with 110 repeated holdouts using 40% of the data as training and 60% as validation set. The models were adjusted for maternal age, pre-pregnancy BMI, ETS exposure during pregnancy, education at enrollment, and parity.*

[b] The mean beta over the 110 repeated holdouts is presented with its 95% confidence interval.

[c] The number of adjusted betas from the 110 repeated holdouts that were positive

Table S15. Mean adjusted association from WQS (positive constraint) binomial model with 110 repeated holdouts between 3rd trimester phthalates mixture and ever allergic rhinitis symptoms at 6-8 years age (N= 489)

Ever allergic rhinitis symptoms [a]	Mean OR and 95% CI [b]	OR > 1 [c]

WQS (b_1)	0.95 (0.85, 1.06)	19/110
WQS*sex (b_{12})	0.91 (0.79, 1.05)	11/110
Betas for males and females		
Females (b_1) [d]	0.95 (0.85, 1.06)	19/110
Males (b_2) [d]	0.87 (0.79, 0.95)	0/110

Abbreviations: WQS, weighted quantile sum; OR, Odds Ratio, CI, confidence interval; BMI, body mass index; ETS, environmental tobacco smoke.

Notes: All chemicals were log_2 transformed to reduce skewness in the distribution of the concentrations.

*[a] The association is derived from a stratified WQS linear regression model allowing for sex-specific weights and including an interaction term WQS*sex. The model was run with 110 repeated holdouts using 40% of the data as training and 60% as validation set. The models were adjusted for maternal age, pre-pregnancy BMI, ETS exposure during pregnancy, education at enrollment, and parity.*

[b] The mean beta over the 110 repeated holdouts is presented with its 95% confidence interval.

[c] The number of adjusted betas from the 110 repeated holdouts that were positive

Table S16. Mean adjusted association from WQS (positive constraint) binomial model with 110 repeated holdouts between 3rd trimester phthalates mixture and current atopic dermatitis symptoms at 6-8 years age (N= 489)

Current atopic dermatitis symptoms[a]	Mean OR and 95% CI [b]	OR > 1 [c]
WQS (b_1)	0.92 (0.66, 1.30)	33/110
WQS*sex (b_{12})	1.08 (0.71, 1.65)	80/110
Betas for males and females		
Females (b_1) [d]	0.92 (0.66, 1.30)	33/110
Males (b_2) [d]	1.00 (0.74, 1.34)	56/110

Abbreviations: WQS, weighted quantile sum; OR, Odds Ratio, CI, confidence interval; BMI, body mass index; ETS, environmental tobacco smoke.

Notes: All chemicals were log_2 transformed to reduce skewness in the distribution of the concentrations.

*[a] The association is derived from a stratified WQS linear regression model allowing for sex-specific weights and including an interaction term WQS*sex. The model was run with 110 repeated holdouts using 40% of the data as training and 60% as validation set. The models were adjusted for maternal age, pre-pregnancy BMI, ETS exposure during pregnancy, education at enrollment, and parity.*

[b] The mean beta over the 110 repeated holdouts is presented with its 95% confidence interval.

[c] The number of adjusted betas from the 110 repeated holdouts that were positive

Table S17. Mean adjusted association from WQS (positive constraint) binomial model with 110 repeated holdouts between 3rd trimester phthalates mixture and current allergic rhinitis symptoms at 6-8 years age (N= 489)

Current allergic rhinitis symptoms [a]	Mean OR and 95% CI [b]	OR > 1 [c]
WQS (b_1)	0.95 (0.85, 1.06)	15/110
WQS*sex (b_{12})	0.94 (0.82, 1.07)	20/110
Betas for males and females		
Females (b_1) [d]	0.95 (0.85, 1.06)	15/110
Males (b_2) [d]	0.89 (0.82, 0.97)	1/110

Abbreviations: WQS, weighted quantile sum; OR, Odds Ratio, CI, confidence interval; BMI, body mass index; ETS, environmental tobacco smoke.

Notes: All chemicals were log_2 transformed to reduce skewness in the distribution of the concentrations.

*[a] The association is derived from a stratified WQS linear regression model allowing for sex-specific weights and including an interaction term WQS*sex. The model was run with 110 repeated holdouts using 40% of the data as training and 60% as validation set. The models were adjusted for maternal age, pre-pregnancy BMI, ETS exposure during pregnancy, education at enrollment, and parity.*

[b] The mean beta over the 110 repeated holdouts is presented with its 95% confidence interval.

[c] The number of adjusted betas from the 110 repeated holdouts that were positive

Table S18. Mean adjusted association from WQS (positive constraint) binomial model with 110 repeated holdouts between 3rd trimester phthalates mixture and Current allergic rhinitis symptoms + itchy watery eyes at 6-8 years age (N=489)

Current allergic rhinitis symptoms + itchy watery eyes [a]	Mean OR and 95% CI [b]	OR > 1 [c]
WQS (b_1)	0.91 (0.77, 1.10)	10/110
WQS*sex (b_{12})	1.10 (0.87, 1.28)	79/110
Betas for males and females		
Females (b_1) [d]	0.91 (0.77, 1.10)	22/110
Males (b_2) [d]	0.96 (0.86, 1.07)	2/110

Abbreviations: WQS (weighted quantile sum), OR (Odds Ratio), CI (confidence interval), BMI (body mass index), ETS (environmental tobacco smoke).
All compounds underwent log2 transformation to decrease skewness in the concentration distribution.
*a) The association is based on a stratified Weighted Quantile Sum (WQS) linear regression model that incorporates sex-specific weights and features an interaction term WQS*sex. The model was executed using 110 repeated holdouts, utilizing 40% of the data for training and 60% for validation. The models were modified for mother age, pre-pregnancy BMI, exposure to environmental tobacco smoke during pregnancy, educational attainment at enrollment, and parity.*
b) The average beta throughout the 110 repeated holdouts is displayed along with its 95% confidence interval.
c) The quantity of corrected betas from the 110 repeated holdouts that were positive.
*d) The adjusted beta for females (b1) represents the beta for the WQS index within the reference group (females = 0), while the adjusted beta for males (b2), the comparison group (males = 1), is determined by the sum of the beta for the WQS*sex interaction term and the beta for the WQS index for the reference group (b2 = b1 + b12).*

Table S19. Association of prenatal phthalate metabolites during the 2nd trimester with atopic outcomes in childhood in the PROGRESS cohort at 4-6 years of age (n=558)

	Risk Ratio (95% Confidence Interval)				
**Metabolites**	Ever atopic dermatitis symptoms	Current atopic dermatitis symptoms	Ever allergic rhinitis symptoms	Current allergic rhinitis symptoms	Current allergic rhinitis symptoms + itchy watery eyes
**High molecular weight**					
**∑DEHP**	0.96 (0.81 – 1.15)	1.00 (0.83 – 1.21)	0.99 (0.93 – 1.06)	0.99 (0.93 – 1.06)	0.96 (0.82 – 1.11)
**MECPTP**	0.84* (0.72 – 0.98)	0.84* (0.71 – 0.98)	0.99 (0.93 – 1.05)	0.99 (0.92 – 1.06)	1.02 (0.88 – 1.19)
**∑DiNP**	0.87 (0.74 – 1.03)	0.92 (0.77 – 1.09)	1.02 (0.95 – 1.09)	1.02 (0.95 – 1.09)	0.97 (0.83 – 1.14)
**MCNP**	0.89 (0.72 – 1.1)	0.91 (0.72 – 1.16)	1.00 (0.92 – 1.08)	1.02 (0.94 – 1.11)	0.91 (0.76 – 1.10)
**MCPP**	0.98 (0.82 – 1.17)	1.00 (0.82 – 1.21)	1.04 (0.97 – 1.12)	1.04 (0.97 – 1.13)	1.00 (0.84 – 1.20)
**MBzP**	0.94 (0.81 – 1.1)	0.96 (0.81 – 1.13)	0.97 (0.92 – 1.03)	0.97 (0.92 – 1.03)	0.96 (0.83 – 1.10)
**Low molecular weight**					
**∑DiBP**	0.96 (0.79 – 1.17)	1.03 (0.84 – 1.27)	1.00 (0.94 – 1.08)	1.01 (0.94 – 1.08)	0.95 (0.81 – 1.13)
**∑DBP**	0.99 (0.84 – 1.16)	0.99 (0.84 – 1.18)	1.03 (0.97 – 1.1)	1.02 (0.96 – 1.09)	0.98 (0.85 – 1.13)

**MEP**	1.05 (0.92 – 1.19)	1.07 (0.93 – 1.23)	1.00 (0.96 – 1.06)	1.01 (0.96 – 1.07)	0.93 (0.81 – 1.07)

Adjusted for maternal age and education at enrollment, parity, pre preganacy BMI, smoke inside
**p<0.05*
Abbreviations: Di-2-ethylhexyl phthalate (DEHP), Diisononyl phthalate (DINP), Diisobutyl phthalate (DIBP), Dibutyl phthalate (DBP), mono-2-ethyl-5-carboxypentyl terephthalate (MECPTP), mono (carboxy-isononyl) phthalate (MCNP),), mono-3-carboxypropyl phthalate (MCPP), monobenzyl phthalate (MBzP), monoethyl phthalate (MEP)

Table S20. Association of prenatal phthalate metabolites during the 2nd trimester with atopic outcomes in childhood in the PROGRESS cohort at 6-8 years of age (n=558)

	**Risk Ratio (95% Confidence Interval)**				
**Metabolites**	Ever atopic dermatitis symptoms	Current atopic dermatitis symptoms	Ever allergic rhinitis symptoms	Current allergic rhinitis symptoms	Current allergic rhinitis symptoms + itchy watery eyes
**High molecular weight**					
**∑DEHP**	0.99 (0.79 – 1.24)	1.02 (0.79 – 1.32)	0.91* (0.84 – 0.99)	0.93 (0.85 – 1.02)	1.00 (0.87 – 1.15)
**MECPTP**	1.02 (0.84 – 1.24)	1.05 (0.83 – 1.33)	0.95 (0.88 – 1.02)	0.99 (0.91 – 1.07)	1.06 (0.94 – 1.20)
**∑DiNP**	0.95 (0.79 – 1.16)	1.01 (0.80 – 1.28)	0.96 (0.87 – 1.05)	0.99 (0.90 – 1.09)	1.08 (0.94 – 1.23)
**MCNP**	0.97 (0.77 – 1.23)	0.96 (0.72 – 1.28)	0.92 (0.83 – 1.03)	0.98 (0.87 – 1.10)	1.15 (0.97 – 1.35)
**MCPP**	0.97 (0.76 – 1.22)	0.96 (0.72 – 1.28)	0.90* (0.82 – 0.99)	0.93 (0.84 – 1.03)	0.97 (0.84 – 1.11)
**MBzP**	0.97 (0.82 – 1.15)	0.97 (0.79 – 1.18)	0.96 (0.90 – 1.04)	1.00 (0.92 – 1.08)	1.02 (0.90 – 1.16)

**Low molecular weight**					
**∑DiBP**	0.95 (0.74 – 1.21)	0.99 (0.74 – 1.33)	0.90* (0.83 – 0.98)	0.94 (0.85 – 1.03)	1.02 (0.89 – 1.17)
**∑DBP**	0.96 (0.77 – 1.11)	0.93 (0.71 – 1.21)	0.94 (0.86 – 1.01)	0.97 (0.89 – 1.05)	0.98 (0.87 – 1.11)
**MEP**	1.00 (0.84 – 1.18)	0.96 (0.76 – 1.17)	1.00 (0.94 – 1.07)	1.01 (0.94 – 1.08)	1.03 (0.92 – 1.16)

Adjusted for maternal age and education at enrollment, parity, pre preganacy BMI, smoke inside
$^{*}p<0.05$

Abbreviations: Di-2-ethylhexyl phthalate (DEHP), Diisononyl phthalate (DINP), Diisobutyl phthalate (DIBP), Dibutyl phthalate (DBP), mono-2-ethyl-5-carboxypentyl terephthalate (MECPTP), mono (carboxy-isononyl) phthalate (MCNP),), mono-3-carboxypropyl phthalate (MCPP), monobenzyl phthalate (MBzP), monoethyl phthalate (MEP)

Table S21. Association of prenatal phthalate metabolites during the 3rd trimester with allergic outcomes in childhood in the PROGRESS cohort at 4-6 years of age (N= 489)

Risk Ratio (95% Confidence Interval)

**Metabolites**	Ever atopic dermatitis symptoms	Current atopic dermatitis symptoms	Ever allergic rhinitis symptoms	Current allergic rhinitis symptoms	Current allergic rhinitis symptoms + itchy watery eyes
**High molecular weight**					
**∑DEHP**	0.88 (0.72 – 1.08)	0.90 (0.73 – 1.10)	1.01 (0.94 – 1.09)	1.03 (0.95 – 1.12)	0.89 (0.72 – 1.09)
**MECPTP**	0.84* (0.72 – 0.97)	0.84* (0.72 – 0.98)	1.00 (0.94 – 1.08)	1.00 (0.94 – 1.09)	0.96 (0.80 – 1.14)
**∑DiNP**	0.91 (0.73 – 1.15)	0.94 (0.74 – 1.20)	0.98 (0.90 – 1.06)	0.97 (0.89 – 1.06)	0.82* (0.68 – 0.98)
**MCNP**	0.899 (0.702 – 1.153)	0.909 (0.695 – 1.190)	1.030 (0.936 – 1.13)	1.05 (0.95 – 1.16)	0.98 (0.77 – 1.24)

**MCPP**	0.96 (0.79 – 1.17)	0.96 (0.77 – 1.18)	1.01 (0.93 – 1.09)	1.02 (0.93 – 1.11)	0.81* (0.67 – 0.99)
**MBzP**	0.95 (0.81 – 1.10)	0.97 (0.82 – 1.13)	0.99 (0.93 – 1.05)	1.00 (0.93 – 1.07)	0.93 (0.79 – 1.11)
**Low molecular weight**					
**∑DiBP**	1.00 (0.82 – 1.23)	1.06 (0.86 – 1.30)	1.01 (0.94 – 1.09)	1.01 (0.93 – 1.10)	0.91 (0.76 – 1.08)
**∑DBP**	0.97 (0.83 – 1.14)	0.96 (0.81 – 1.14)	0.99 (0.93 – 1.06)	1.00 (0.93 – 1.07)	0.86 (0.72 – 1.02)
**MEP**	1.02 (0.90 – 1.16)	1.08 (0.95 – 1.23)	1.01 (0.96 – 1.07)	1.01 (0.95 – 1.07)	0.99 (0.87 – 1.13)

Adjusted for maternal age and education at enrollment, parity, pre preganacy BMI, smoke inside

**p<0.05*
Abbreviations Di-2-ethylhexyl phthalate (DEHP), Diisononyl phthalate (DINP), Diisobutyl phthalate (DIBP), Dibutyl phthalate (DBP), mono-2-ethyl-5-carboxypentyl terephthalate (MECPTP), mono (carboxy-isononyl) phthalate (MCNP),), mono-3-carboxypropyl phthalate (MCPP), monobenzyl phthalate (MBzP), monoethyl phthalate (MEP)

Table S22. Association of prenatal phthalate metabolites during the 3rd trimester with atopic outcomes in childhood in the PROGRESS cohort at 6-8 years of age (n= 489)

	Risk Ratio (95% Confidence Interval)				
**Metabolites**	Ever atopic dermatitis symptoms	Current atopic dermatitis symptoms	Ever allergic rhinitis symptoms	Current allergic rhinitis symptoms	Current allergic rhinitis symptoms + itchy watery eyes
**High molecular weight**					
**∑DEHP**	0.83 (0.64 – 1.08)	0.90 (0.66 – 1.23)	0.95 (0.86 –1.04)	0.94 (0.85 – 1.04)	0.94 (0.80 – 1.10)
**MECPTP**	0.82 (0.64 – 1.06)	0.89 (0.65 – 1.21)	0.94 (0.86 – 1.01)	0.93 (0.85 – 1.01)	0.96 (0.84 – 1.10)

**∑DiNP**	0.87 (0.62 – 1.22)	0.98 (0.66 – 1.46)	0.92 (0.84 – 1.01)	0.92 (0.83 – 1.02)	0.95 (0.80 – 1.12)
**MCNP**	0.75 (0.53 – 1.07)	0.83 (0.54 – 1.27)	0.91 (0.81 – 1.02)	0.92 (0.81 – 1.04)	0.93 (0.76 – 1.13)
**MCPP**	1.02 (0.73 – 1.42)	1.15 (0.79 – 1.67)	0.91* (0.83 – 1.00)	0.90* (0.82 – 1.00)	0.89 (0.76 – 1.05)
**MBzP**	0.90 (0.73 – 1.11)	0.95 (0.74 – 1.23)	0.96 (0.89 – 1.04)	0.97 (0.90 – 1.05)	0.94 (0.83 – 1.07)
**Low molecular weight**					
**∑DiBP**	0.89 (0.65 – 1.21)	0.98 (0.66 – 1.45)	0.95 (0.87 – 1.04)	0.97 (0.88 – 1.07)	0.92 (0.79 – 1.08)
**∑DBP**	0.97 (0.74 – 1.27)	0.96 (0.68 – 1.34)	0.94 (0.87 – 1.02)	0.95 (0.87 – 1.03)	0.91 (0.79 – 1.04)
**MEP**	0.99 (0.81 – 1.22)	0.97 (0.78 – 1.21)	0.97 (0.91 – 1.04)	0.98 (0.91 – 1.05)	0.99 (0.89 – 1.09)

Adjusted for maternal age and education at enrollment, parity, pre preganacy BMI, smoke inside
**p<0.05*
Abbreviations Di-2-ethylhexyl phthalate (DEHP), Diisononyl phthalate (DINP), Diisobutyl phthalate (DIBP), Dibutyl phthalate (DBP), mono-2-ethyl-5-carboxypentyl terephthalate (MECPTP), mono (carboxy-isononyl) phthalate (MCNP),), mono-3-carboxypropyl phthalate (MCPP), monobenzyl phthalate (MBzP), monoethyl phthalate (MEP)

Table S23. Association of 2nd trimester phthalate metabolite concentrations and allergic outcomes stratified by child sex

Metabolite	Outcome	Male N=276	Female N=282	
		Risk Ratio (95% Confidence Interval)		P-value for interaction
MCNP	Ever atopic dermatitis symptoms at 4-6 years	1.16 (0.88 – 1.54)	0.67 (0.48 – 0.92)*	0.01
MCNP	Current atopic dermatitis symptoms at 4-6 years	1.21 (0.87 – 1.66)	0.70 (0.50 – 0.98) *	0.02
∑DiBP	Current allergic rhinitis symptoms + itchy watery eyes at 4-6 years	0.81 (0.65 – 1.00)*	1.16 (0.92 – 1.47)	0.02
∑DBP	Ever allergic rhinitis symptoms at 4-6 years	0.98 (0.91 – 1.06)	1.11 (1.01 – 1.23)*	0.05
∑DBP	Current allergic rhinitis symptoms at 4-6 years	0.97 (0.89 – 1.05)	1.11 (1.00 – 1.23)*	0.03

MCPP	Current atopic dermatitis symptoms at 6-8 years	1.34 (0.86 – 2.08)*	0.69 (0.47 – 1.02)	0.03
